# Supplementary material for: Bladder cancer therapy using a conformationally fluid tumoricidal peptide complex
Source: Nat Commun. 2021 Jun 8;12:3427. doi: 10.1038/s41467-021-23748-y (PMC8187399; doi:10.1038/s41467-021-23748-y)
Supplement: Supplementary file 1 — Supplementary Information [file 41467_2021_23748_MOESM1_ESM.pdf]

Supplementary Information

Bladder cancer therapy using a conformationally fluid tumoricidal peptide complex

Antonín Brisuda, James C.S. Ho, Pancham S. Kandiyal, Justin T-Y. Ng, Ines Ambite, Daniel S.C. Butler, Jaromir Háček, Murphy Lam Yim Wan, Thi Hien Tran, Aftab Nadeem, Tuan Hiep Tran, Anna Hastings, Petter Storm, Daniel L. Fortunati, Parisa Esmaeili, Hana Novotna, Jakub Horňák, Y.G. Mu, K. H. Mok, Marek Babjuk and Catharina Svanborg.

Supplementary References

(*1*) H. Edelhoch, The properties of thyroglobulin. I. The effects of alkali. *J Biol Chem* **235**, 1326-1334 (1960).

(*2*) J. White *et al.*, The aggregation of "native" human serum albumin. *Eur Biophys J* **44**, 367-371 (2015).

(*3*) R. Parker, T. R. Noel, G. J. Brownsey, K. Laos, S. G. Ring, The nonequilibrium phase and glass transition behavior of beta-lactoglobulin. *Biophys J* **89**, 1227-1236 (2005).

(*4*) D. K. Wilkins *et al.*, Hydrodynamic radii of native and denatured proteins measured by pulse field gradient NMR techniques. *Biochemistry* **38**, 16424-16431 (1999).

(*5*) R. J. Hunter, *Introduction to Modern Colloid Science*. (Oxford University Press, 1993).

(*6*) Tosoh Bioscience GmbH, "SEC: Size Exclusion Chromatography" (2017).

(*7*) D.J. States, R.A. Haberkorn, D.J. Ruben, A two-dimensional nuclear overhauser experiment with pure absorption phase in four quadrants. *J Magn Reson (1969)* **48**, 286-292 (1982).

(*8*) M.D. Pelta, H. Barjat, G.A. Morris, A.L. Davis, S.J. Hammond. Pulse sequences for high-resolution diffusion-ordered spectroscopy (HR-DOSY). *Magn Reson Chem.* **36**, 706-714 (1998).

(*9*) J.A. Jones, D.K. Wilkins, L.J. Smith, C.M. Dobson. Characterisation of protein unfolding by NMR diffusion measurements. *J Biomol NMR* **10**, 199-203 (1997).

Supplementary Fig. 1

a

```
alpha1       1 KQFTKAELSQLLKDIDGYGGIALPELIATMFHTSGYDTQ-----------      39
                                                .:|:|..
sar1alpha    1 -------------------------------MAGWDIFGWFRDVLASLG      18

Identity:       2/55 ( 3.6%)
Similarity:     4/55 ( 7.3%)
Gaps:          48/55 (87.3%)
Score: 10.0
```

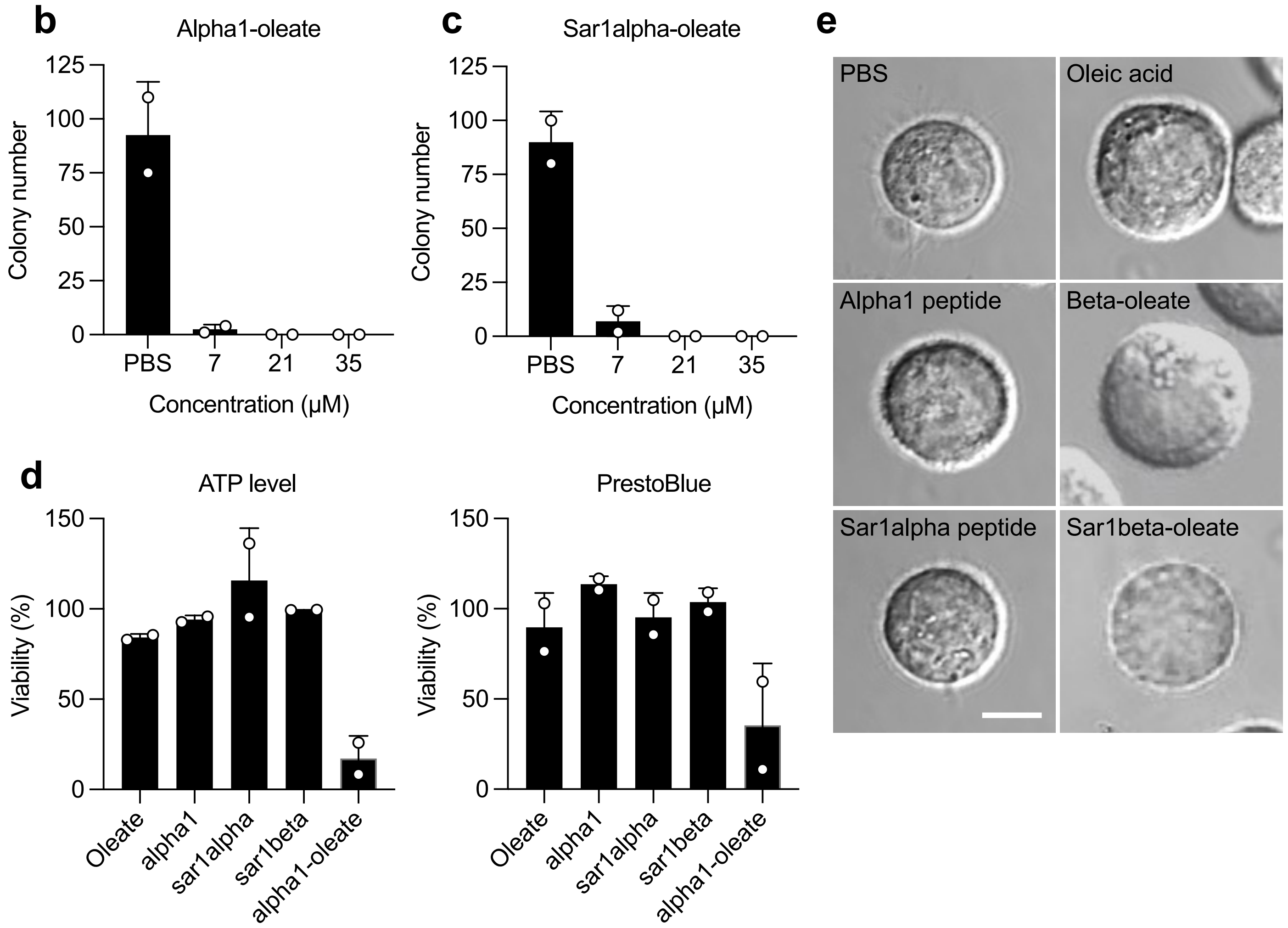

Supplementary Fig. 1. a, Sequence alignment of alpha1 and sar1alpha peptides. EMBOSS Needle pairwise sequence alignments showing a lack of homology between alpha1 and sar1alpha, which are derived from proteins with unrelated functions and low identity. **b,c,** Quantification for colony assay with increasing doses of alpha1-oleate (**b**) (images in **Fig. 1e**) or sar1alpha-oleate (**c**) (images in **Supplementary Fig. 2e**). Mean ± SD, *n* = 2 independent experiments. **d**, The naked alpha1 and sar1alpha peptides lacked tumoricidal activity, as did the sar1beta peptide and oleate, quantified as a reduction in ATP levels and by PrestoBlue fluorescence (compared to control). Alpha1-oleate was included as a positive control. Lung carcinoma cells, 35 μM, 1 hour. Mean ± SD, *n* = 2 independent experiments. **e**, The naked alpha peptides (35 μM), oleic acid (175 μM) or beta peptides complexes (35 μM) did not trigger membrane blebbing in A549 lung carcinoma cells (10 min). Representative of 2 independent experiments. Scale bar = 10 μm.

Supplementary Fig. 2

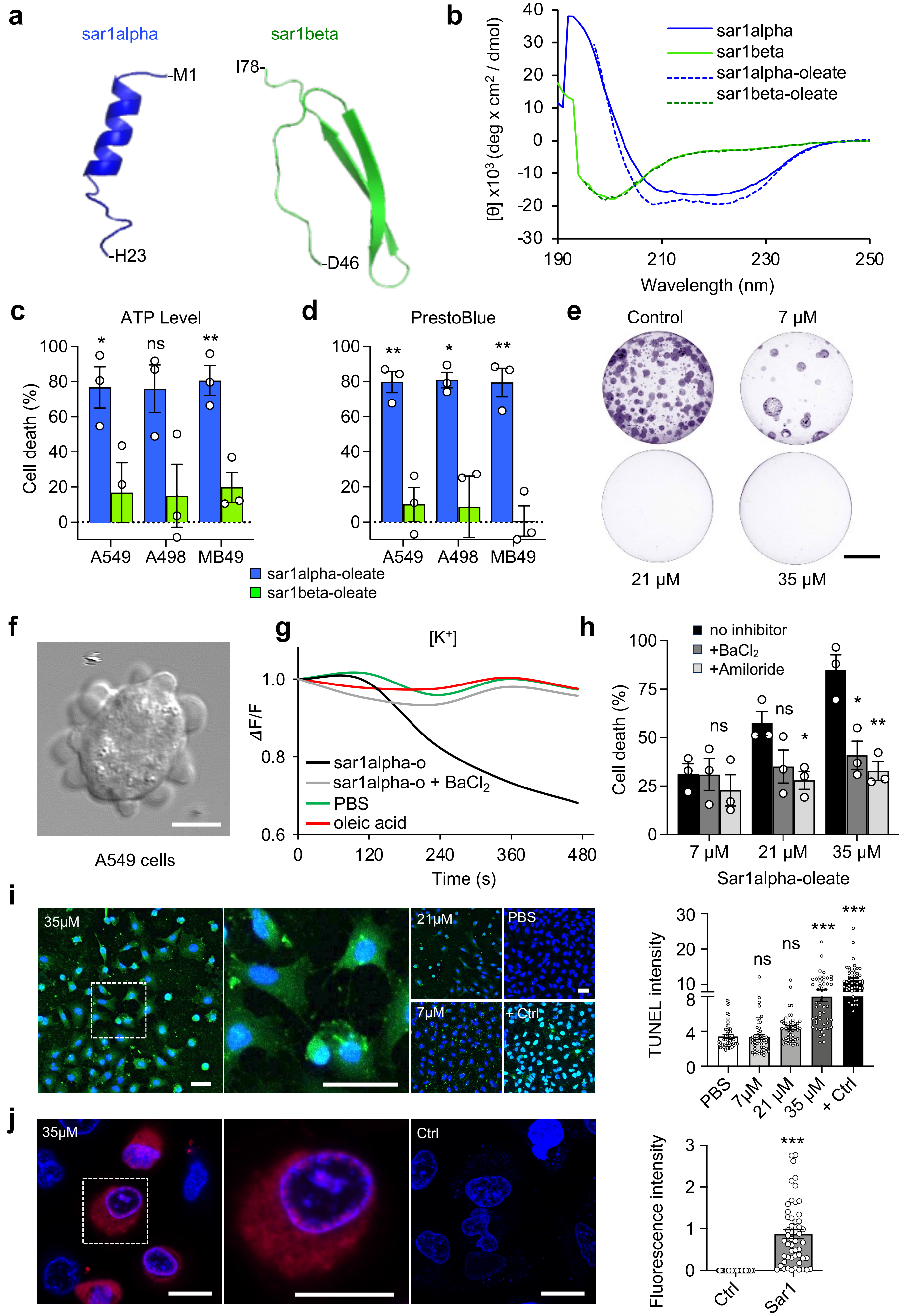

Supplementary Fig. 2. Tumoricidal activity of two non-homologous alpha-helical peptide-oleate complexes.

a, Structural models of sar1alpha and sar1beta peptides generated by I-TASSER. **b**, Far-UV circular dichroism spectra of synthetic sar1alpha, sar1beta peptide, and their respective peptide-oleate complexes. **c, d,** Cell death of human lung (A549), kidney (A498) and murine bladder (MB49) carcinoma cells, quantified as a reduction in (**c**) ATP levels (P = 0.044 for A549 and 0.007 for MB49, sar1alpha-oleate compared to sar1beta-oleate) or (**d**) PrestoBlue fluorescence (P = 0.004 for A549, 0.017 for A498 and 0.002 for MB49, sar1alpha-oleate compared to sar1beta-oleate). Cells were treated with the sar1alpha1-oleate complex (blue) or the sar1beta-oleate complex (green), (3 h, 35 μM, % of control). **e,** Colony assay showing dose-dependent long-term effects sar1alpha-oleate (MB49, 10 days). Scale bar = 5 mm. **f,** Sar1alpha-oleate triggers rapid membrane blebbing in A549 lung carcinoma cells (35 μM, 10 min). Scale bar = 10 μm. Representative of 3 independent experiments. **g,** K^+ efflux in A549 lung carcinoma cells exposed to sar1alpha-oleate and inhibition with $BaCl_2$. **h**, Inhibition of cell death by the ion flux inhibitors Amiloride and $BaCl_2$ (100 μM), measured by PrestoBlue fluorescence (P = 0.015 for 35 μM with $BaCl_2$, 0.019 for 21 μM with Amiloride and 0.005 for 35 μM with Amiloride, % of control). **i**, DNA strand breaks detected by TUNEL staining in sar1alpha-oleate treated A549 lung carcinoma cells (n = 50 cells per group). Scale bars = 20 μm. **j**, AlexaFluor568-labeled sar1alpha-oleate (red) is internalized by A549 lung carcinoma cells. Nuclei are counterstained with DAPI (blue) (n = 52 cells per group). Scale bars = 10 μm. Data are presented as means ± S.E.Ms from three independent experiments, * P < 0.05, ** P < 0.01, *** P < 0.001, analyzed by unpaired two-tailed t-test (**c, d, h, j**) and two-tailed ANOVA using Dunnett's correction (**i**).

Supplementary Fig. 3

a

SE-HPLC standard curve (R_H)

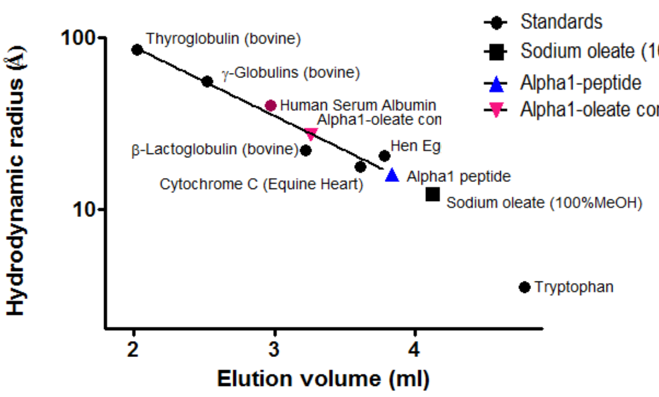

Protein Standard	No. of residues	R_H (Å)	Elution vol. (mL)	Ref.
Thyroglobulin	2769	85.8	2.02	(*1*)
γ-Globulin	1364	56	2.52	(*2*)
Human serum albumin (HSA)	603	40	2.97	(*2*)
β-Lactoglobulin	162	22	3.22	(*3*)
Hen egg white lysozyme	129	20.5	3.78	(*4*)
Cytochrome C	105	17.8	3.61	(*4*)
Tryptophan	n/a	3.5	4.77	(*5*)

b

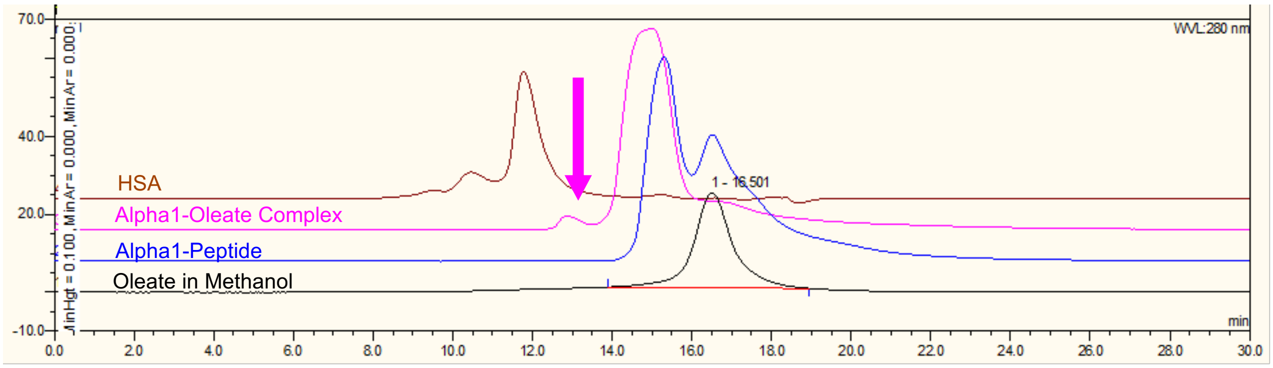

Supplementary Fig. 3. Size-exclusion HPLC (SE-HPLC) of the alpha1 peptide and the alpha1-oleate complex. Calibration standards and samples were injected onto a TSKgel Super SW3000 HPLC column (4.6 mm x 30 cm, Particle size 4 µm, Pore size 25 nm, Tosoh Bioscience) eluted with 0.05 M sodium phosphate buffer pH 7.0 containing 0.1 M Na_2SO_4 at a flow rate of 0.25 mL/min and detection at 280 nm. The chromatography was performed on a Dionex Ultimate HPLC 3000 Standard System running Chromeleon 6 software (Dionex, Thermo Scientific). **a,** The standard calibration curve was generated with the proteins indicated, including human serum albumin (HSA), which has a hydrodynamic radius (R_H) of 40 Å. The alpha1 peptide and the alpha1-oleate complex were injected and their R_H values were determined to be 16.1 Å and 27.4 Å, respectively (colored symbols), which agree well with the results from diffusion-ordered NMR. The R_H vs. elution volume linearity of the standard calibration curve is known to vanish after approximately 3.8 mL of elution volume (*6*). As a result, the R_H of oleate in methanol eluent (a solvent which ensures that the fatty acid is monomeric) will be less than what is estimated from the standard curve (12.3 Å). The retention times of small R_H-analytes are closely reproduced regardless of eluent, be it aqueous buffer or methanol. **b**, Chromatograms of HSA, alpha1-oleate complex, and alpha1-peptide in aqueous buffer eluent, and oleate in methanol eluent (colour-coded as given in panel A above). By definition, size-exclusion chromatography chemistry tends to strip complexes of non-covalent, weakly bound molecules, and as a result, the intact complex peak gradually decreases in amount as the sample passes through the column (shown by the arrow).

Supplementary Fig. 4

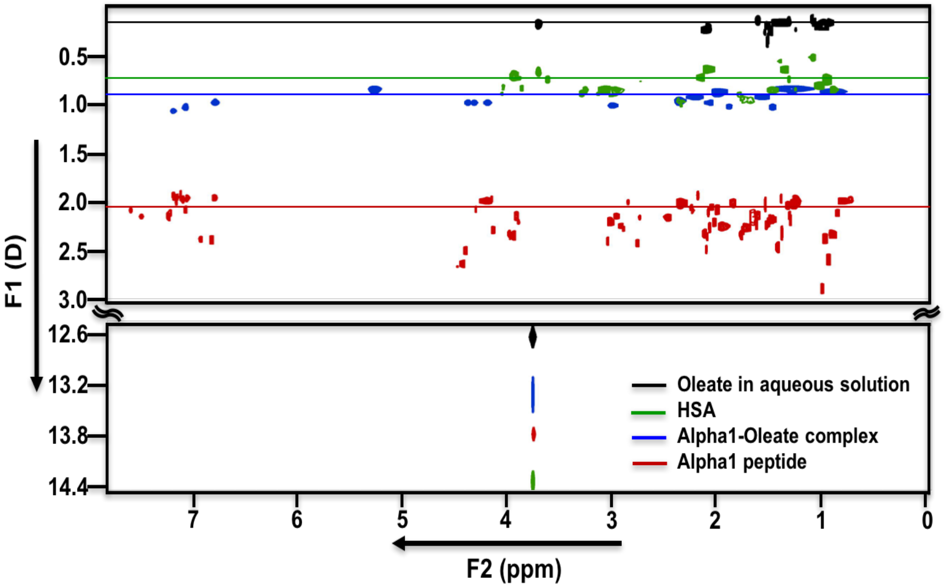

Supplementary Fig. 4. Diffusion-ordered NMR spectroscopy (DOSY) of the alpha1 peptide and the alpha1-oleate complex. Diffusion-ordered spectroscopy (DOSY) measurements were performed at 293 K. Samples were prepared in 50 mM phosphate buffer at pH 7.4. The DgscteSL_dpfgsc DOSY pulse program was used, which consists of gradient compensated stimulated echo with spin lock using the excitation sculpting solvent suppression method (*7*). A spectral window of 13020 Hz was used, with an acquisition time of 2.46 s with a relaxation delay of 3 s. The FIDs were collected with 32000 complex data points with 64 scans. Logarithmically the gradient pulse strength was increased from 3 % to 86 % of the maximum strength of 32767 G/cm in 60 steps. A diffusion time (Δ) of 100 ms and bipolar half-sine-shaped gradient pulses (δ) of 5 ms were applied. 1,4-Dioxane, which is known to behave independently of protein concentration and the folded state of the protein, was used as an internal chemical shift reference and hydrodynamic radius calibration reference (3.75 ppm; R_H = 2.12 Å) (*8, 9*). DOSY processing was performed using a two-component fit with discrete approach, which further processed using non-uniform gradients approach. Three replicate acquisitions were given for each sample, and the resulting diffusion coefficient (D) values calculated. For alpha1 peptide the average D value for was 2.162 m^2/s and 14.10 m^2/s for 1,4-dioxane. In case of alpha1-oleate complex the average D value was 0.986 m^2/s for complex and 13.61 m^2/s for 1.4-dioxane. Color-coding of the samples are identical to Extended Data Fig. 9, and the calculated R_H are as follows: alpha1 peptide R_H = 13.82 ± 0.447 Å, alpha1-oleate complex R_H = 29.3 ± 0.606 Å, human serum albumin R_H = 40.9 ± 1.44 Å, oleate in aqueous solution R_H = 104.3 ± 7.22 Å, oleate in methanol (spectrum not shown) R_H = 5.58 ± 0.0649 Å. Note that the D (diffusion coefficient) values for 1,4-dioxane are slightly variable dependent upon the co-solute (lower panel where D is between 14.4 and 12.6), which rightly reflects the different solution micro-environment conditions that both solutes are mutually experiencing for each sample.

Supplementary Fig. 5

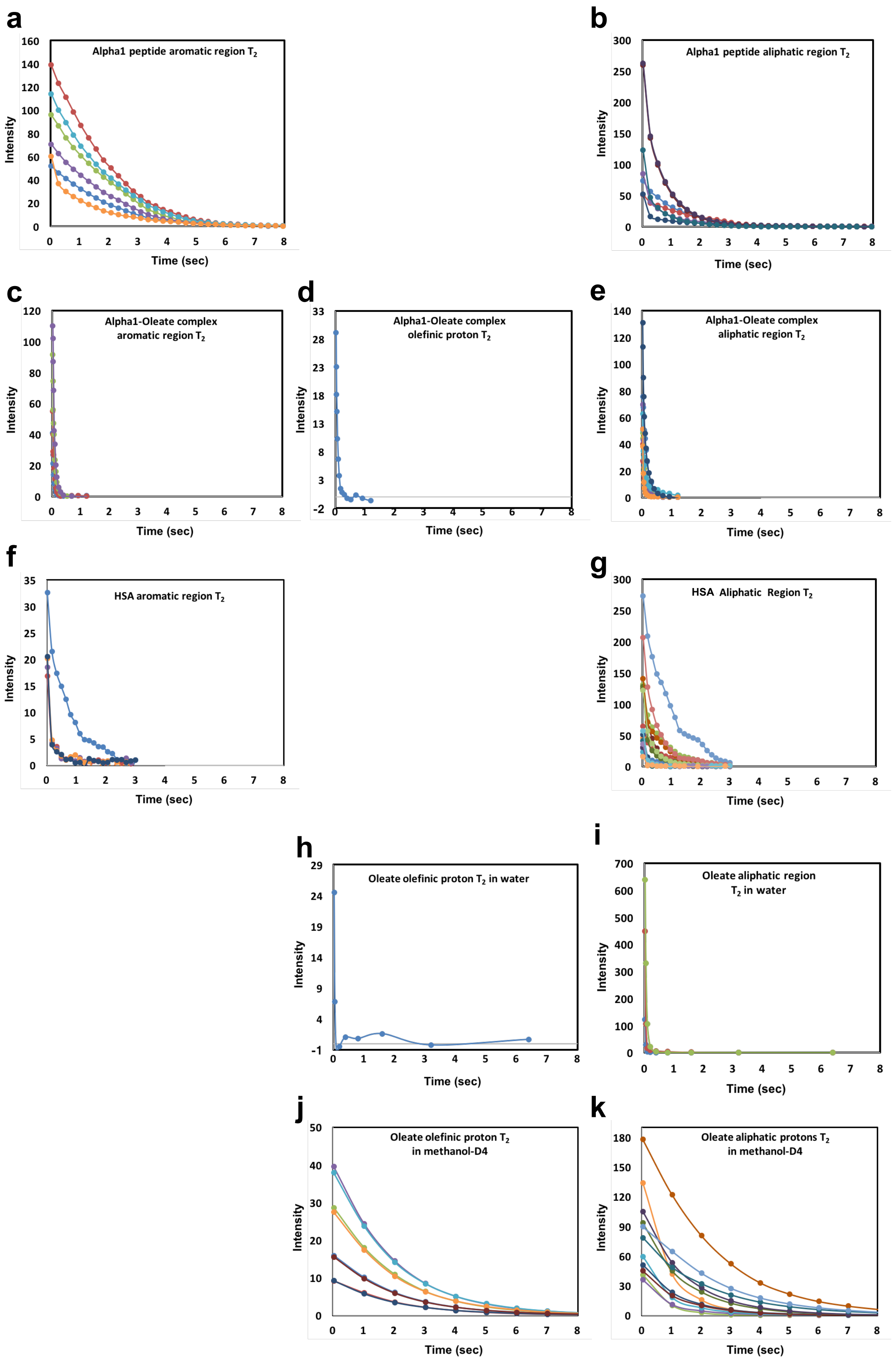

1H Chemical Shift (ppm)

Supplementary Fig. 5. T_2 relaxation ^{1}H NMR curves of the alpha1 peptide and the alpha1-oleate complex.

For T_2 relaxation measurements, the standard CPMGT2 pulse sequence was used to run the experiments with 15 relaxations delays, which were chosen logarithmically for different maximum T_2 time intervals: 8 s (alpha1 peptide), 1.2 s (alpha1-oleate complex), 3.0 s (HSA), 7.0 s (oleate in aqueous solution), and 10 s (oleate in methanol) respectively. The data were acquired with 32000 complex points with baseline correction of 4. The T_2 analyses were performed on VNMRJ version 4.0 (Agilent Technologies) software by exponential fitting of these values with their corresponding intensity. All other NMR parameters were kept constant for all samples throughout the experiments. The experiments were acquired at sample temperature of 293 K. The data are presented in Supplementary Table 1. To guide in visualization of the large differences in signal decay, all relaxation curve plots are extended to 8 s, **a**, Alpha1 peptide aromatic region, **b**, alpha1 peptide aliphatic region, **c**, alpha1-oleate complex aromatic region, **d**, olefinic protons of the bound oleate in the alpha1-oleate complex, **e**, alpha1-oleate complex aliphatic region, **f**, human serum albumin aromatic region, **g**, human serum albumin alphatic region, **h**, olefinic protons of oleate in aqueous solution, **i**, oleate in aqueous solution aliphatic range, **j**, olefinic protons of oleate in methanol, **k**, oleate in methanol aliphatic range.

Supplementary Fig. 6

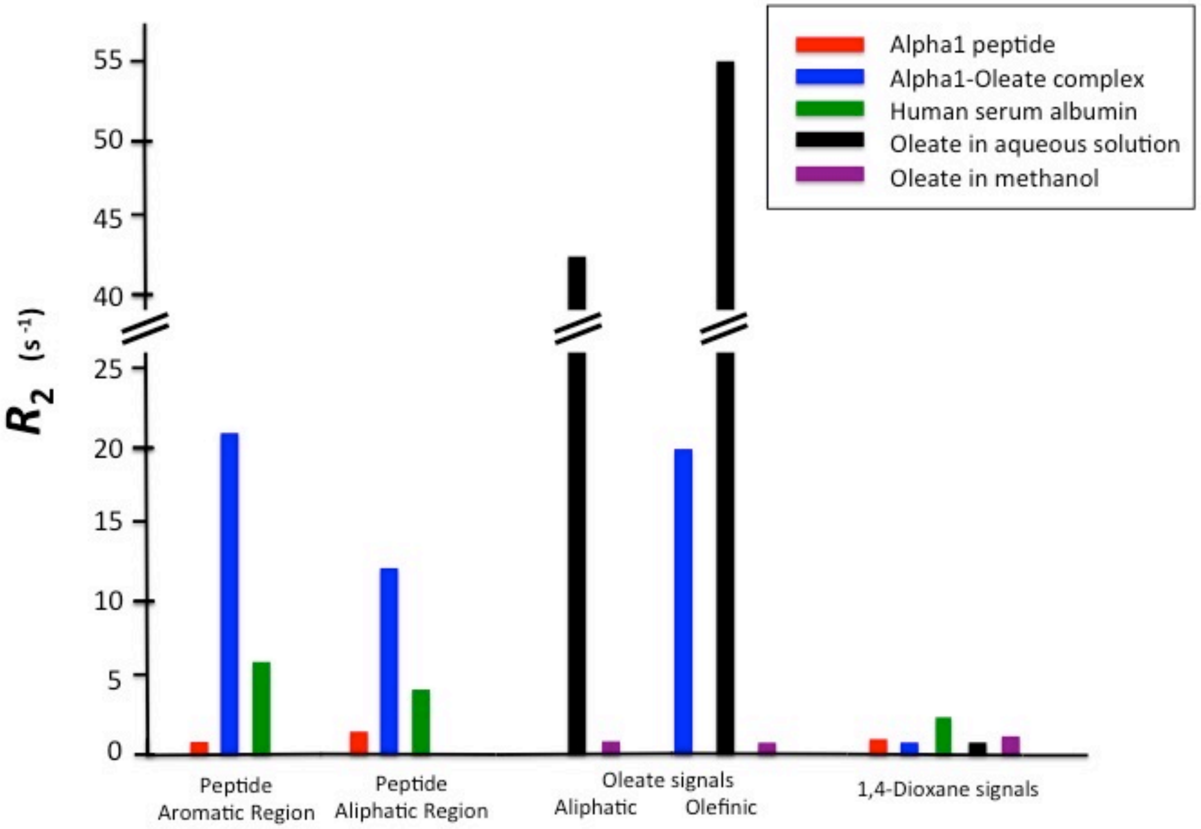

Supplementary Fig. 6. Average R_2 transverse relaxation rates of the alpha1 peptide and the alpha1-oleate complex.

Average values are from Supplementary Table 1. Note that the 1,4-dioxane R_2 relaxation rates are relatively consistent regardless of the co-solute or solvent, suggesting that the remarkably different relaxation behavior of the alpha1 peptide and the alpha1-oleate complex are due to significant differences in intrinsic motions and not on the solvent environment.

Supplementary Fig. 7

a

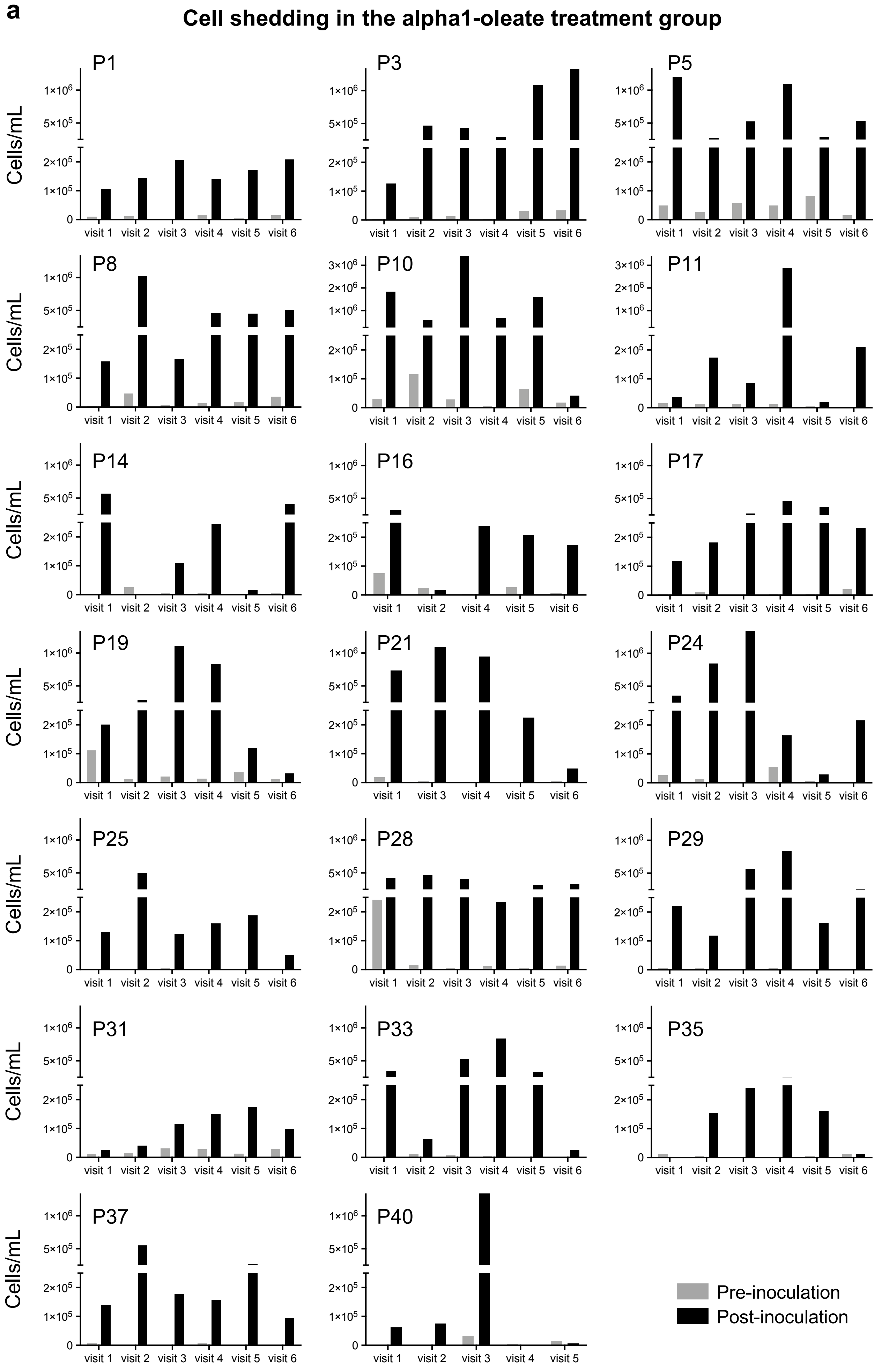

b

Cell shedding in the placebo group

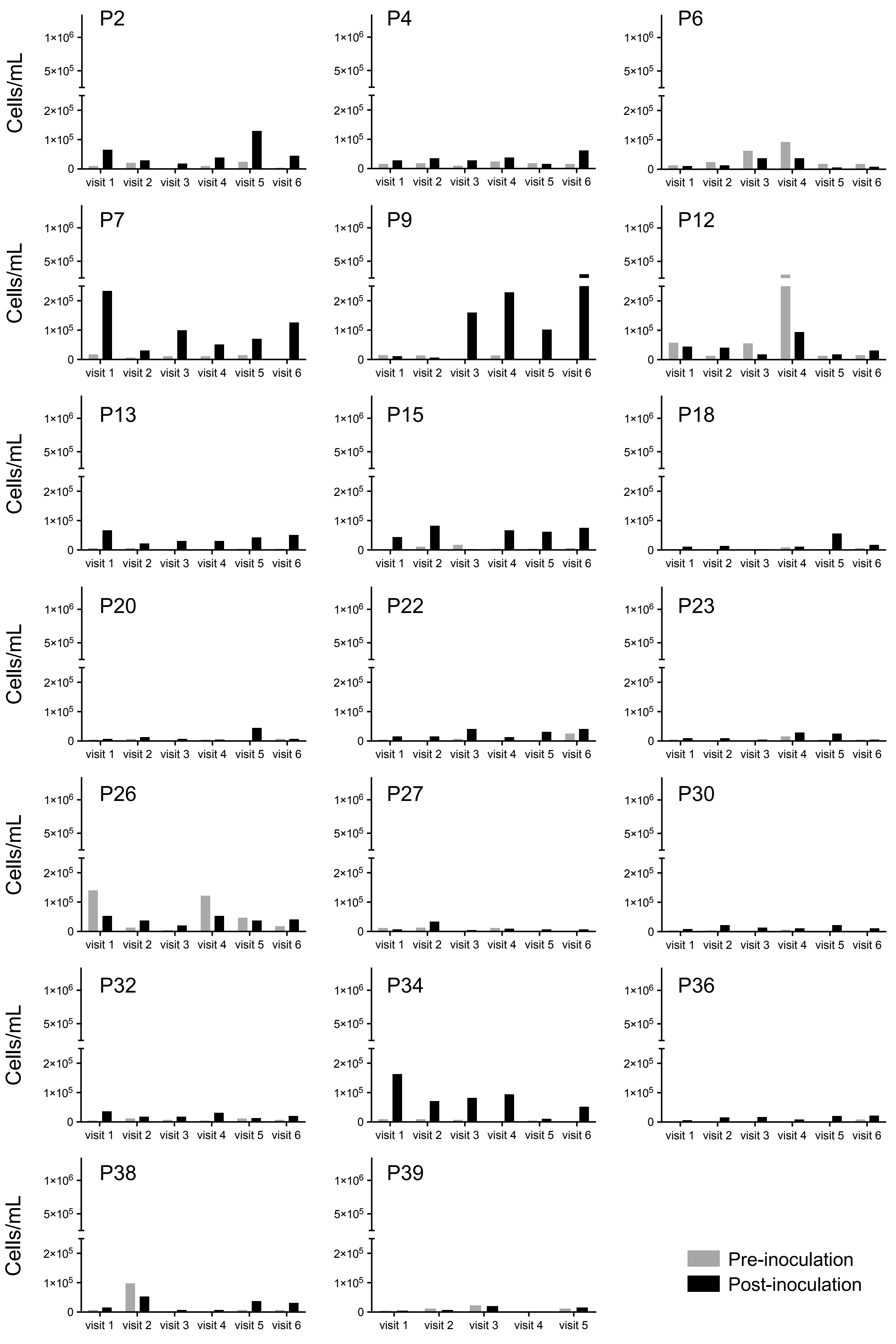

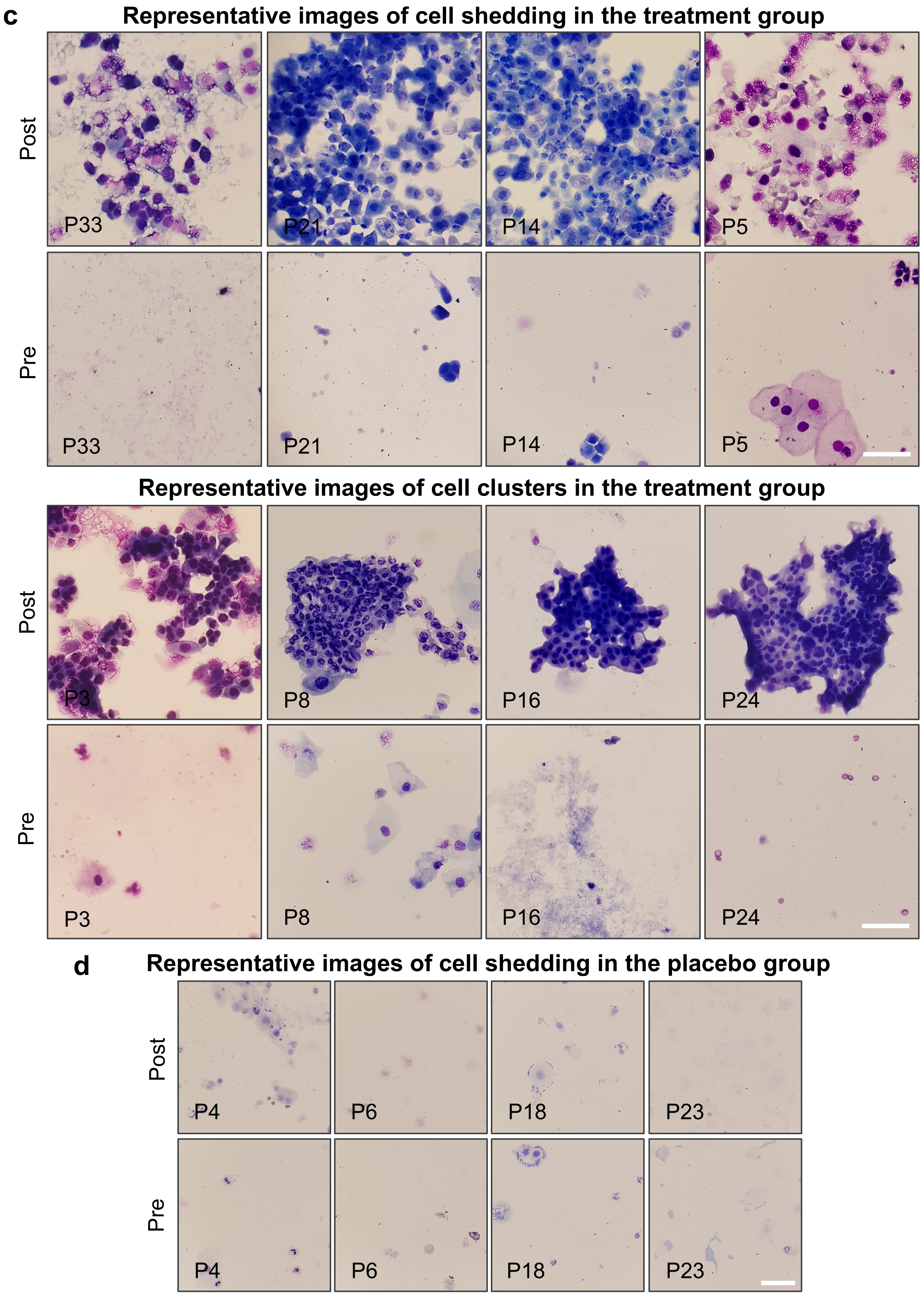

Supplementary Fig. 7. Shedding of tumor cells following intravesical instillation of alpha1-oleate. a, b, Histogram of cell numbers in individual patients before and about two hours after each instillation of alpha1-oleate (**a**, 1.7 mM, 30 mL) and placebo (**b,** PBS, 30 mL). **c**, Representative images from patient urine, illustrating the increase in cell numbers after alpha1-oleate instillation, compared to pre-inoculation samples. Quantifications are shown in **Fig. 5b,e**. Scale bars = 50 μm. **d**, Representative images from patient urine, illustrating the lack of response in patients receiving placebo, compared to pre-inoculation samples. Scale bar = 50 μm. Cytospin slides stained with H&E.

Supplementary Fig. 8

Evidence of tumor cell apoptosis - TUNEL

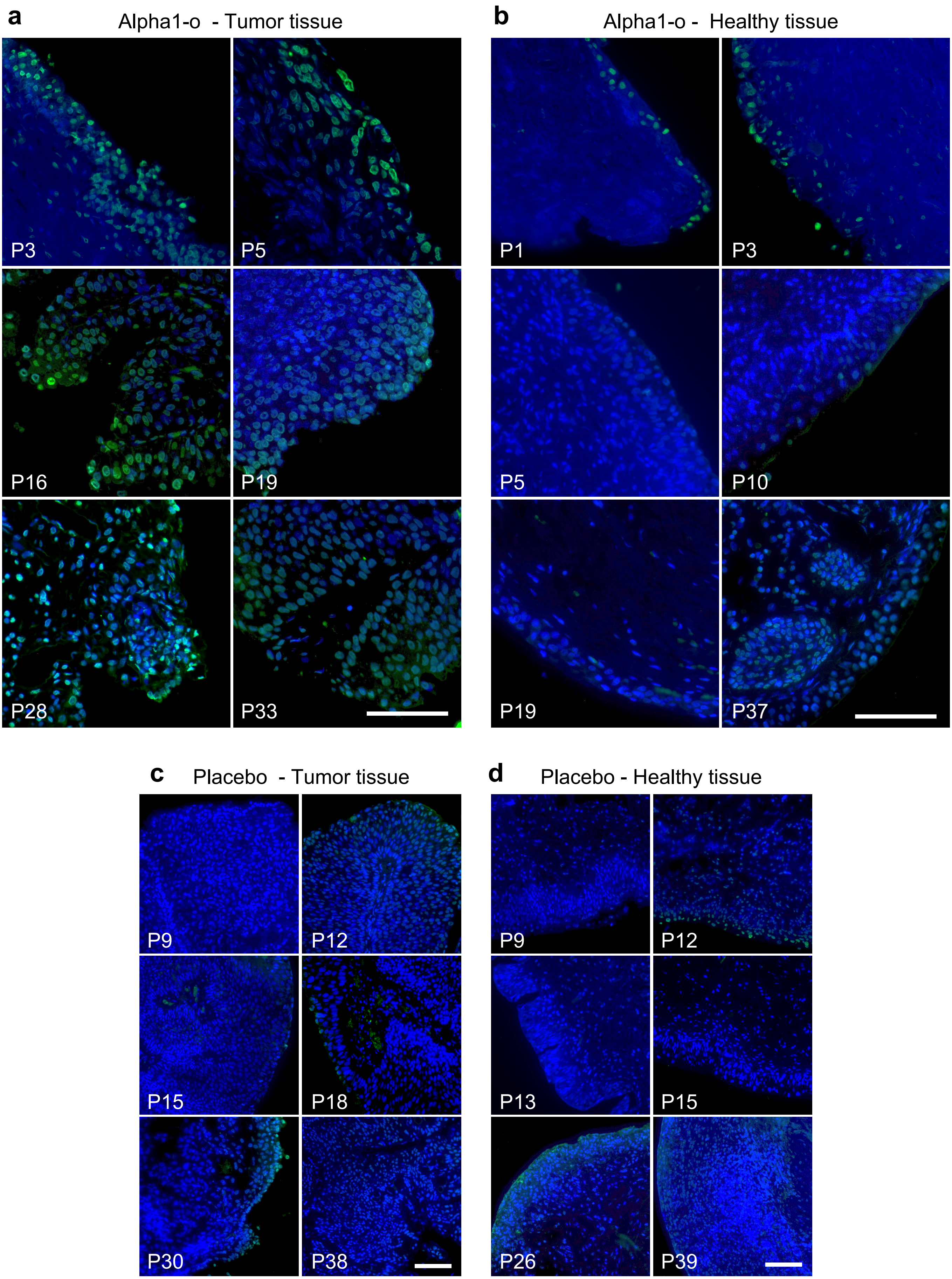

Supplementary Fig. 8. Apoptotic response to alpha1-oleate treatment.
DNA strand breaks detected by TUNEL staining in tissue sections from tumor biopsies or healthy tissue. **a**, Representative images from tumor biopsies in individual patients receiving alpha1-oleate. **b**, Representative images from healthy tissue biopsies in patients receiving alpha1-oleate. Healthy tissue receiving alpha1-oleate instillations, **c**, Representative images from tumor biopsies in patients receiving placebo. **d**, Representative images from healthy tissue biopsies in patients receiving placebo. Quantifications are shown in **Fig. 6c**. Scale bars = 200 μm.

Supplementary Fig. 9

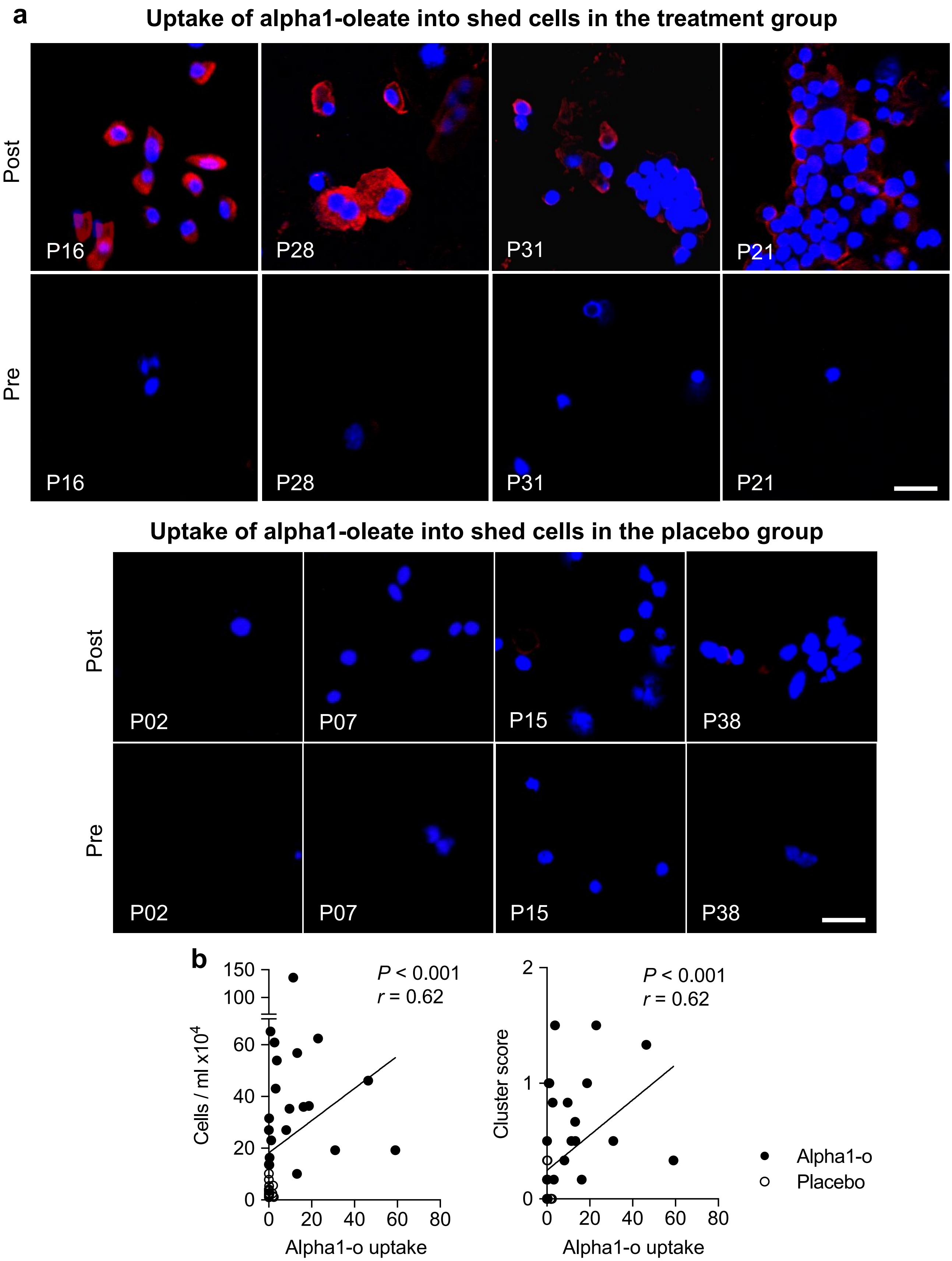

Supplementary Fig. 9. Uptake of alpha1-oleate.

a, Representative images of alpha1-oleate uptake by tumor cells, visualized by staining with anti-alpha1 peptide antibodies. Staining was detected in the treatment but not in the placebo group. Scale bars = 20 µm. **b**, The alpha1-oleate uptake by tumor cells was significantly correlated with cell shedding in individual patients (95% CI 0.3759 - 0.7852) and with the tumor cell cluster scores (95% CI 0.3770 - 0.7857). Spearman correlation, two-tailed, approximate *P*-value, *n* = 20 per group.

Supplementary Fig. 10

a

Cancer genes, Tumor (Alpha1-o vs. Placebo)

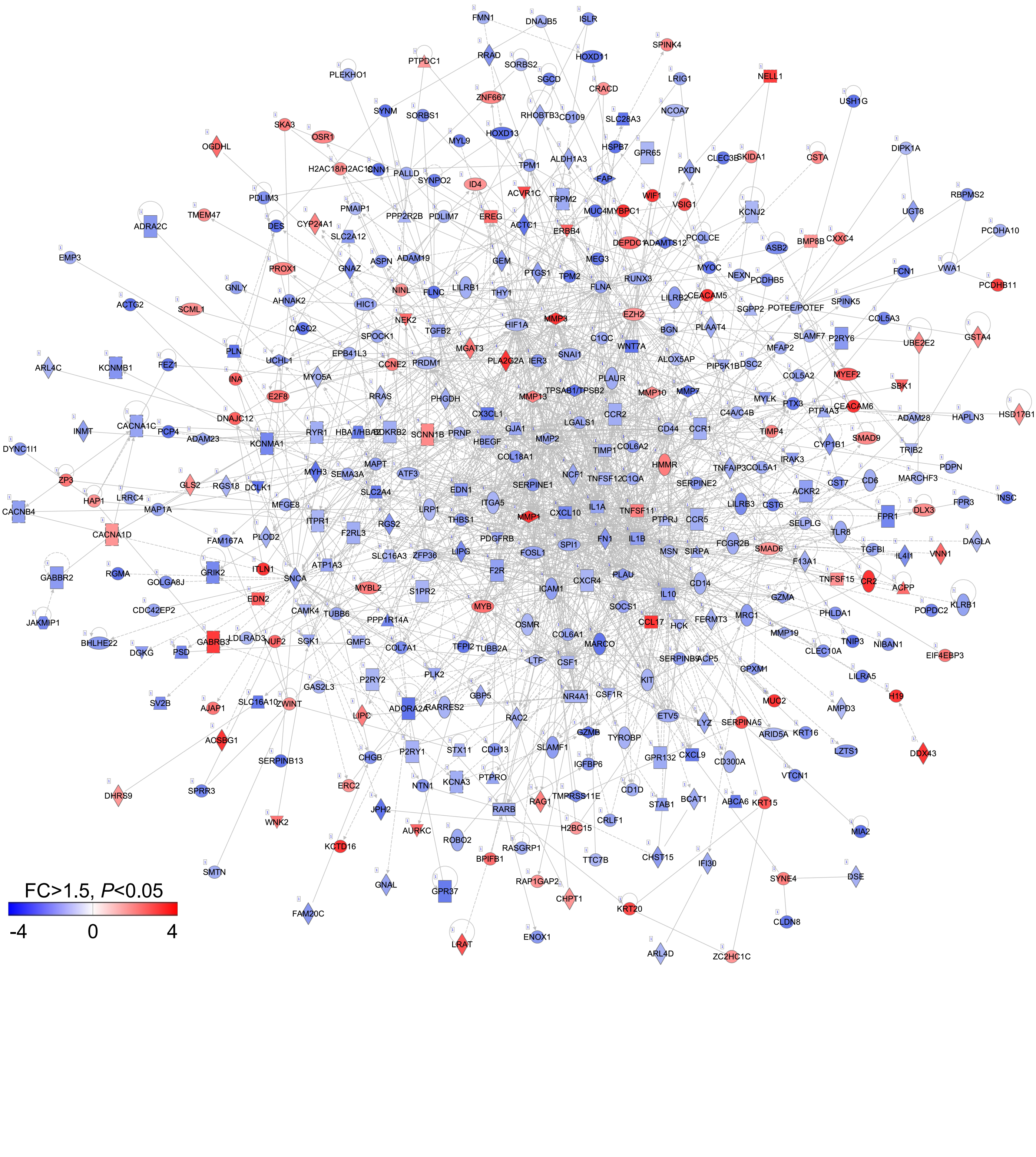

b

Rho family GTPases pathway, Tumor (Alpha1-o vs. Placebo)

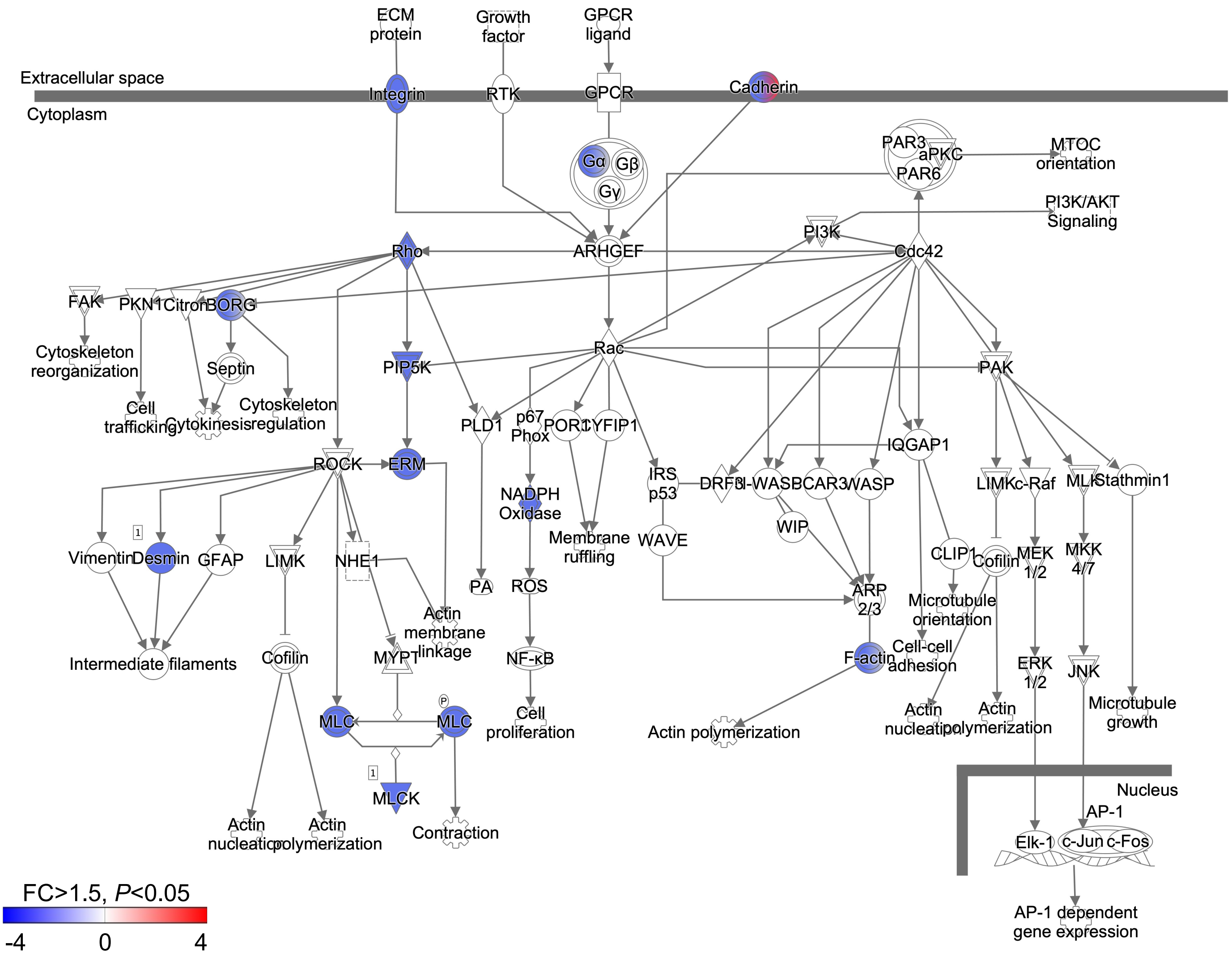

c

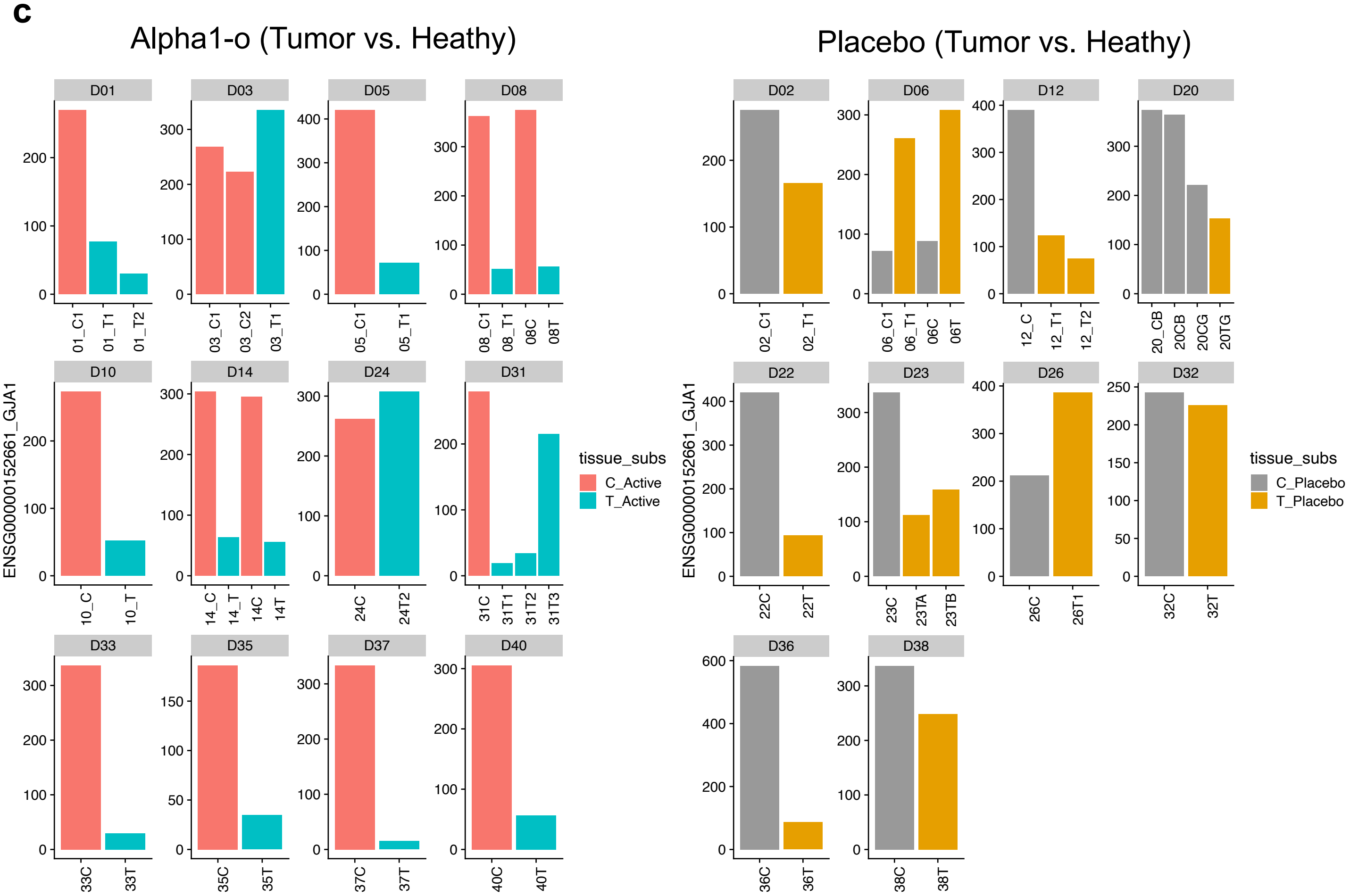

Supplementary Fig. 10. RNA sequencing analysis.
RNA extracted from tumor and healthy biopsies was analyzed by RNA sequencing. Gene expression data were analyzed by IPA software and significantly regulated genes (cut-off FC 1.5, $P < 0.05$) were identified. Red: upregulated genes, blue: down-regulated genes **a**, Genes in the cancer network (IPA) were largely inhibited (399/572 genes) in treated tumor tissue compared to placebo. Major interacting nodes included *HIF1A* (hypoxia-inducible factor involved in angiogenesis and cellular metabolism), *PLAUR* (urokinase receptor and plasminogen activator involved in metastasis), *IL1B* (cytokine that may play role in tumorgenesis due to its immunomodulatory properties), *FN1* (fibronectine involved in carcinoma development enhancing tumorigenicity and tumor survival), *SERPINE1* (plasminogen activator inhibitor), *MMP2* (collagenase involved in cancer cell invasion and metastasis), *CSF1* (macrophage-colony stimulating factor involved in cancer cell invasion and metastasis) and *GJA1* (connexin-43, associated with cancer development and metastasis). **b**, Inhibition of Rho family GTPases and downstream signaling in the treatment group compared to placebo. **c**, About 20 genes were differentially expressed in tumor tissue compared to healthy tissue exclusively in the treatment group (adjusted *P* value < 0.05). Inhibition of GJA1/Connexin 43 expression in the treatment compared to the placebo group.

Supplementary Fig. 11

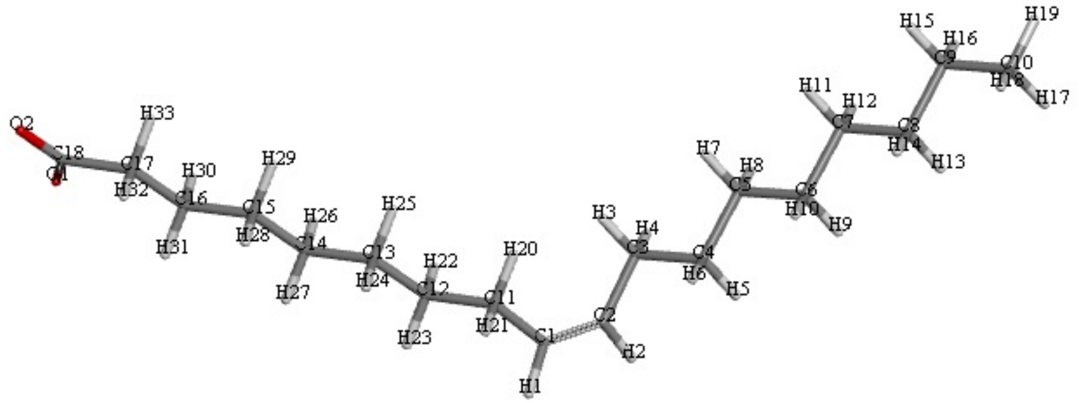

Supplementary Fig. 11. Structure of Oleate molecule with atom labels.
Carbon atoms are shown as grey sticks, hydrogen atoms as white sticks, and oxygen atoms as red sticks.

Supplementary Table 1. ***R_2*** **transverse relaxation rates of alpha1- peptide and alpha1-oleate complexes.**

Chemical shift region	Aromatics region protons					Olefinic protons					Aliphatic region protons				
**Compound**	**δ (ppm)**	**T_2* (sec)**	**Error**	**Avg. T_2 and SD**	**R_2 (1/T_2)**	**δ (ppm)**	**T_2* (sec)**	**Error**	**Avg. T_2 and SD**	**R_2 (1/T_2)**	**δ (ppm)**	**T_2* (sec)**	**Error**	**Avg. T_2 and SD**	**R_2 (1/T_2)**
**Alpha1 peptide**	7.422<br>7.412<br>7.403<br>7.361<br>7.309<br>7.083	1.884<br>1.943<br>2.020<br>1.916<br>1.958<br>1.210	0.037<br>0.042<br>0.060<br>0.048<br>0.039<br>0.071	T_2 = 1.821<br>SD = 0.339	R_2 = 0.549<br>SD =<br>2.949						3.982<br>3.831<br>3.740<br>3.549<br>2.122<br>2.021<br>1.446<br>0.950<br>0.947<br>0.939<br>0.917	1.228<br>1.574<br>0.543<br>2.115<br>2.102<br>1.179<br>0.323<br>0.581<br>0.629<br>0.602<br>0.366	0.013<br>0.049<br>0.033<br>0.035<br>0.092<br>0.024<br>0.035<br>0.017<br>0.020<br>0.019<br>0.020	T_2 = 1.022<br>SD = 0.663	R_2 = 0.978<br>SD = 1.50
**Alpha1 peptide complex**	7.200<br>7.088<br>6.809<br>6.800	0.029<br>0.044<br>0.056<br>0.060	0.001<br>0.003<br>0.003<br>0.002	T_2 = 0.047<br>SD = 0.013	R_2 = 21.27<br>SD =<br>76.93	5.251	0.050	0.002	0.050	R_2 = 20	4.680<br>4.370<br>2.344<br>2.170<br>2.153<br>1.970<br>1.942<br>0.904<br>0.833	0.119<br>0.160<br>0.141<br>0.062<br>0.066<br>0.056<br>0.045<br>0.020<br>0.075	0.007<br>0.014<br>0.003<br>0.006<br>0.003<br>0.004<br>0.005<br>0.001<br>0.006	T_2 = 0.083<br>SD= 0.046	R_2= 12.04<br>SD=21.73
**HSA**	8.447<br>7.472<br>7.310<br>7.290<br>7.249<br>7.119<br>7.094	0.635<br>0.133<br>0.141<br>0.134<br>0.117<br>0.117<br>0.091	0.046<br>0.019<br>0.018<br>0.020<br>0.018<br>0.015<br>0.007	T_2 = 0.195<br>SD = 0.194	R_2= 5.128<br>SD=5.15						3.900<br>3.663<br>3.645<br>3.226<br>3.197<br>2.947<br>2.662<br>2.213<br>2.031<br>1.612<br>1.314<br>1.263<br>1.182<br>1.173<br>1.164<br>1.156<br>1.003<br>0.930<br>0.600	0.243<br>0.483<br>0.564<br>0.215<br>0.178<br>0.045<br>0.128<br>0.307<br>0.160<br>0.064<br>0.085<br>0.068<br>0.408<br>0.911<br>0.398<br>0.255<br>0.121<br>0.092<br>0.100	0.033<br>0.042<br>0.043<br>0.024<br>0.007<br>0.006<br>0.004<br>0.005<br>0.012<br>0.007<br>0.011<br>0.005<br>0.041<br>0.056<br>0.015<br>0.013<br>0.010<br>0.006<br>0.017	T_2 = 0.253<br>SD= 0.219	R_2= 3.952<br>SD=4.56

**Oleate in aqueous solution**						5.34	0.018	0.002	0.018	R_2 = 55.03	2.025<br>1.294<br>0.881	0.017<br>0.017<br>0.039	0.0003<br>0.0005<br>0.0012	T_2 = 0.024<br>SD= 0.013	R_2= 41.66<br>SD=76.92
**Oleate in methanol**						5.340<br>5.337<br>5.335<br>5.332<br>5.330<br>5.328<br>5.325<br>5.323	2.064<br>2.115<br>2.046<br>1.979<br>2.017<br>2.058<br>2.011<br>2.036	0.023<br>0.039<br>0.024<br>0.015<br>0.019<br>0.024<br>0.015<br>0.016	T_2 = 2.040<br>SD =0.040	R_2 = 0.490<br>SD=25.0	2.151<br>2.141<br>2.131<br>2.031<br>2.022<br>2.014<br>2.005<br>1.330<br>1.321<br>1.316<br>1.305<br>1.296<br>1.288<br>1.286<br>0.902<br>0.893<br>0.884	0.243<br>0.309<br>0.252<br>0.693<br>0.757<br>0.729<br>0.564<br>0.892<br>0.882<br>0.875<br>1.301<br>1.298<br>1.424<br>1.504<br>2.207<br>2.453<br>2.566	0.007<br>0.006<br>0.007<br>0.030<br>0.013<br>0.009<br>0.014<br>0.024<br>0.025<br>0.017<br>0.015<br>0.021<br>0.019<br>0.011<br>0.025<br>0.038<br>0.074	T_2 = 1.115<br>SD= 0.727	R_2= 0.896<br>SD=1.375

Supplementary Table 2. Per residue and average secondary structures propensities for alpha1 peptides.

	Secondary Structure Propensities					
	alpha1-oleate			alpha1		
Residue Number	Helix	Sheet	Others	Helix	Sheet	Others
LYS 1	0.00	0.00	1.00	0.00	0.00	1.00
GLN 2	0.00	0.02	0.98	0.00	0.00	1.00
PHE3	0.00	0.75	0.25	0.00	0.48	0.52
THR4	0.00	0.18	0.82	0.00	0.58	0.42
LYS 5	0.48	0.01	0.51	0.21	0.25	0.55
ALA 6	0.59	0.18	0.24	0.21	0.12	0.67
GLU7	0.59	0.16	0.25	0.31	0.10	0.59
LEU 8	0.70	0.00	0.30	0.44	0.00	0.56
SER 9	0.66	0.00	0.34	0.34	0.00	0.66
GLN 10	0.62	0.00	0.38	0.23	0.06	0.72
LEU 11	0.52	0.00	0.48	0.12	0.36	0.52
LEU 12	0.37	0.00	0.63	0.03	0.35	0.63
LYS 13	0.03	0.00	0.97	0.00	0.01	0.99
ASP 14	0.03	0.06	0.91	0.17	0.06	0.76
ILE 15	0.16	0.07	0.77	0.26	0.34	0.40
ASP 16	0.37	0.02	0.60	0.27	0.00	0.73
GLY 17	0.38	0.02	0.60	0.27	0.00	0.73
TYR 18	0.28	0.02	0.70	0.07	0.34	0.59
GLY 19	0.64	0.00	0.36	0.02	0.06	0.92
GLY20	0.64	0.00	0.36	0.24	0.00	0.75
ILE 21	0.62	0.02	0.36	0.24	0.14	0.62
ALA22	0.49	0.04	0.48	0.24	0.32	0.43
LEU23	0.68	0.08	0.24	0.00	0.19	0.81
PRO24	0.69	0.06	0.25	0.14	0.13	0.74
GLU 25	0.94	0.00	0.06	0.74	0.00	0.26
LEU 26	0.86	0.00	0.14	0.75	0.00	0.25
ILE 27	0.61	0.00	0.39	0.81	0.00	0.19
ALA28	0.42	0.00	0.58	0.75	0.00	0.25
THR29	0.26	0.01	0.73	0.55	0.00	0.45
MET30	0.10	0.01	0.89	0.42	0.01	0.57
PHE31	0.06	0.89	0.04	0.44	0.01	0.55
HIS 32	0.06	0.18	0.77	0.35	0.01	0.64
THR33	0.02	0.00	0.98	0.12	0.00	0.88
SER 34	0.00	0.00	1.00	0.07	0.00	0.93
GLY 35	0.07	0.00	0.93	0.01	0.01	0.98
TYR 36	0.07	0.02	0.91	0.05	0.00	0.95
ASP 37	0.07	0.01	0.92	0.05	0.01	0.94
THR 38	0.00	0.15	0.84	0.05	0.00	0.95
GLN39	0.00	0.00	1.00	0.00	0.00	1.00
Peptide Average	0.34	0.08	0.59	0.23	0.10	0.67

Supplementary Table 3. Per residue and average secondary structure propensities for sar1alpha peptides.

	Secondary Structure Propensities					
	sar1alpha-oleate			sar1alpha		
Residue Number	Helix	Sheet	Others	Helix	Sheet	Others
MET 1	0.00	0.00	1.00	0.00	0.00	1.00
ALA 2	0.01	0.01	0.98	0.02	0.32	0.66
GLY 3	0.10	0.00	0.90	0.03	0.31	0.66
TRP 4	0.53	0.00	0.47	0.06	0.35	0.60
ASP 5	0.65	0.00	0.35	0.10	0.31	0.59
ILE 6	0.70	0.00	0.30	0.09	0.33	0.58
PHE 7	0.74	0.00	0.26	0.08	0.32	0.60
GLY 8	0.78	0.00	0.22	0.10	0.37	0.54
TRP 9	0.76	0.00	0.24	0.09	0.45	0.46
PHE 10	0.81	0.00	0.19	0.09	0.44	0.47
ARG 11	0.86	0.00	0.14	0.11	0.35	0.54
ASP 12	0.88	0.00	0.12	0.11	0.67	0.22
VAL 13	0.66	0.00	0.34	0.14	0.69	0.17
LEU 14	0.51	0.00	0.49	0.14	0.39	0.47
ALA 15	0.89	0.00	0.11	0.14	0.08	0.78
SER 16	0.84	0.00	0.16	0.11	0.01	0.88
LEU 17	0.55	0.01	0.44	0.10	0.35	0.56
GLY 18	0.46	0.00	0.54	0.08	0.36	0.56
LEU 19	0.18	0.00	0.82	0.15	0.36	0.49
TRP 20	0.31	0.01	0.68	0.20	0.35	0.45
ASN 21	0.31	0.00	0.69	0.17	0.36	0.47
LYS 22	0.23	0.00	0.77	0.12	0.31	0.58
HIS 23	0.00	0.00	1.00	0.00	0.00	1.00
Peptide Average	0.51	0.00	0.49	0.10	0.32	0.58

Supplementary Table 4. Contact probabilities between alpha1- or sar1alpha- side chain protons and olefinic protons.

**alpha1-oleate**		**sar1alpha-oleate**	
**Residue Number**	**Contact Probability**	**Residue Number**	**Contact Probability**
LYS1	0.57	MET1	0.78
GLN2	0.18	ALA2	0.58
PHE3	0.83	GLY3	0.69
THR4	0.07	TRP4	0.81
LYS5	0.81	ASP5	0.13
ALA6	0.13	ILE6	0.93
GLU7	0.32	PHE7	0.93
LEU8	0.97	GLY8	0.35
SER9	0.09	TRP9	0.90
GLN10	0.34	PHE10	0.99
LEU11	0.99	ARG11	0.71
LEU12	0.96	ASP12	0.10
LYS13	0.28	VAL13	0.57
ASP14	0.33	LEU14	0.99
ILE15	0.93	ALA15	0.12
ASP16	0.72	SER16	0.56
GLY17	0.33	LEU17	0.96
TYR18	0.95	GLY18	0.09
GLY19	0.79	LEU19	0.95
GLY20	0.69	TRP20	0.86
ILE21	0.90	ASN21	0.14
ALA22	0.78	LYS22	0.63
LEU23	1.00	HIS23	0.62
PRO24	0.53		
GLU25	0.65		
LEU26	0.98		
ILE27	0.38		
ALA28	0.13		
THR29	0.67		
MET30	0.97		
PHE31	0.98		
HIS32	0.06		
THR33	0.16		
SER34	0.08		
GLY35	0.08		
TYR36	0.66		
ASP37	0.07		
THR38	0.06		
GLN39	0.08		

Supplementary Table 5. Contact probabilities between aromatic protons and olefinic protons for alpha1-oleate and sar1alpha-oleate.

**alpha1-oleate**		**sar1alpha-oleate**	
**Residue Number**	**Contact Probability**	**Residue Number**	**Contact Probability**
PHE3	0.83	TRP4	0.80
TYR18	0.93	PHE7	0.93
PHE31	0.98	TRP9	0.89
HIS32	0.05	PHE10	0.99
TYR36	0.59	TRP20	0.85
		HIS23	0.59
Aromatic Residues Average	0.68	Aromatic residues Average	0.84

Supplementary Table 6. Patient enrolment.

Inclusion Criteria

- Patient with non-muscle invasive papillary bladder cancer (NMIBC) based on cystoscopy appearance, on the waiting list for TURB.
- Negative pregnancy test in women of childbearing potential.
- Appropriate methods of contraception in women of childbearing potential during study.
- Patients should be able to keep the content of the bladder for at least one hour.

Exclusion Criteria

- Patient with a previous history of muscle invasive bladder cancer.
- Patient with a history of NMIBC with an interval shorter than 6 months after previous TURB.
- Previous intravesical Bacillus Calmette-Guerin (BCG) immunotherapy in the last 12 months.
- Previous intravesical chemotherapy in the last 12 months.
- Participants with any other cancer diagnosis within the last 5 years (except of skin basaliomas).
- Acute urinary tract infection
- Participants with prior radiotherapy or systemic chemotherapy.
- Participants receiving any other investigational agent or non-marketed product one month prior to Visit 1 and during the trial.
- Any concurrent illness that may render a participant ineligible or limit compliance with study requirements.
- Previously enrolled in this trial.

Supplementary Table 7. Summary of demographic data.

Treatment	Parameter	Mean (SD)	Median (Min, Max)
Placebo	Age (years)	63.7 (12.50)	61.0 (41.0, 86.0)
	BMI (kg/m^2)	28.6 (4.96)	27.8 (22.3, 42.8)
	Height (cm)	174.5 (7.94)	173.0 (162.0, 187.0)
	Weight (kg)	87.3 (16.56)	85.5 (60.0, 128.0)
Active	Age (years)	66.5 (11.41)	68.0 (45.0, 90.0)
	BMI (kg/m^2)	27.6 (4.42)	27.0 (19.8, 35.8)
	Height (cm)	175.2 (9.41)	174.0 (154.0, 187.0)
	Weight (kg)	85.7 (19.93)	84.0 (47.0, 125.0)

There was no significant difference between the active and placebo group

Supplementary Table 8. Summary of adverse events by disease group.

	Active (N=20) N (%), E	Placebo (N=20) N (%), E
**Overall**	12 (60.0), 13	11 (55.0), 16
**Renal and urinary disorders**	8 (40.0), 8	6 (30.0), 6
Dysuria	3 (15.0), 3	3 (15.0), 3
Haematuria	2 (10.0), 2	3 (15.0), 3
Micturition urgency	2 (10.0), 2	-
Urinary retention	1 (5.0), 1	-
**Infections and infestations**	4 (20.0), 4	6 (30.0), 7
Urinary tract infection	4 (20.0), 4	4 (20.0), 4
Bacteriuria	-	1 (5.0), 1
Erysipelas	-	1 (5.0), 1
Urosepsis*	-	1 (5.0), 1
**Blood and lymphatic system disorders**	-	1 (5.0), 1
Thrombocytopenia*	-	1 (5.0), 2
**Cardiac disorders**	-	1 (5.0), 1
Tachyarrhythmia	-	1 (5.0), 1
**Nervous system disorders**	-	1 (5.0), 1
Transient ischaemic attack*	-	1 (5.0), 2
**Skin and subcutaneous tissue disorders**	1 (5.0), 1	-
Dermatitis allergic	1 (5.0), 1	-

There was no significant difference between the active and placebo group.

*Serious adverse events.

One subject experienced urosepsis requiring hospitalization and interruption of study drug administration but after appropriate treatment, the patient recovered fully and completed the remaining instillations and TURB. The second subject experienced two transient ischemic attacks with thrombocytopenia, which were defined as medically important events related to underlying disease, but unrelated to the treatment or the procedure. The patient recovered and completed the study. There were no AEs leading to patient withdrawal from the trial or discontinuation of the investigational product.

Supplementary Table 9. Summary of pathology evaluation.

	Active N (%)	Placebo N (%)
**Tumor grade (WHO 1973)**		
Missing		4 (20.0)
Papillary carcinoma, grade 1	4 (20.0)	6 (30.0)
Papillary carcinoma, grade 2	14 (70.0)	7 (35.0)
Papillary carcinoma, grade 3	2 (10.0)	3 (15.0)
**Tumor grade (WHO 2004/2016)**		
Missing		4 (20.0)
High-grade	5 (25.0)	4 (20.0)
Low-grade	15 (75.0)	12 (60.0)
**Tumor stage**		
Ta	14 (70.0)	12 (75.0)
T1	5 (25.0)	4 (25.0)
T2	1 (5.0)	0

Supplementary Table 10. Atom labels, corresponding atom types and partial charges for Oleate molecule.

Atom	Atom Type	Partial Charge	Mass
C1	c2	-0.27428	12
H1	ha	0.13725	1
C2	c2	-0.31519	12
H2	ha	0.13628	1
C3	c3	0.16858	12
H3	hc	0.00472	1
H4	hc	0.00472	1
C4	c3	-0.03521	12
H5	hc	0.00887	1
H6	hc	0.00887	1
C5	c3	-0.01429	12
H7	hc	-0.01022	1
H8	hc	-0.01022	1
C6	c3	0.11368	12
H9	hc	-0.0319	1
H10	hc	-0.0319	1
C7	c3	-0.02086	12
H11	hc	-0.00536	1
H12	hc	-0.00536	1
C8	c3	-0.00953	12
H13	hc	-0.00306	1
H14	hc	-0.00306	1
C9	c3	0.16132	12
H15	hc	-0.02877	1
H16	hc	-0.02877	1
C10	c3	-0.2317	12
H17	hc	0.04852	1
H18	hc	0.04852	1
H19	hc	0.04852	1
C11	c3	0.14538	12
H20	hc	0.00707	1
H21	hc	0.00707	1
C12	c3	-0.0165	12
H22	hc	0.00624	1
H23	hc	0.00624	1
C13	c3	0.00803	12
H24	hc	-0.02199	1
H25	hc	-0.02199	1
C14	c3	0.05504	12
H26	hc	-0.02545	1
H27	hc	-0.02545	1
C15	c3	-0.01683	12

H28	hc	-0.01424	1
H29	hc	-0.01424	1
C16	c3	0.11605	12
H30	hc	-0.0235	1
H31	hc	-0.0235	1
C17	c3	-0.15332	12
H32	hc	-0.01286	1
H33	hc	-0.01286	1
C18	c	0.89604	12
O1	o	-0.8473	16
O2	o	-0.8473	16

Supplementary Note 1

CONFIDENTIAL

Clinical Trial Protocol

A Randomized Placebo controlled Phase I/II Study Evaluating the Safety and Efficacy of α1H in adult patients with non-muscle invasive bladder cancer awaiting transurethral surgery

ICH GCP statement: *The clinical trial will be conducted in compliance with the clinical trial protocol, GCP and the applicable regulatory requirement(s).*

**Hamlet Pharma AB**	**Trial ID:**	**HP002-001**
	**Date:**	**15-Jan-2019**
	**Version:**	**2.2**

THIS DOCUMENT CONTAINS TRADE SECRETS, AND/OR COMMERCIAL INFORMATION, DELIVERED IN CONFIDENCE AND RELIANCE THAT SUCH INFORMATION WILL NOT BE COPIED OR MADE AVAILABLE TO ANY THIRD PARTY WITHOUT THE WRITTEN CONSENT OF HAMLET PHARMA AB.

1 Clinical Trial Protocol Statement

1.1 Approval Statement Hamlet Pharma AB

The trial protocol has been approved by the following person as presented on the last page of this document.

Professor Catharina Svanborg, MD, PhD

Medical Responsible / Hamlet Pharma AB

1.2 Approval Statement International Coordinating Investigator

The international coordinating investigator approves the clinical trial protocol and consolidated clinical trial protocol(s) comprising any subsequent amendment(s).

Professor Marek Babjuk, MD, PhD

International coordinating investigator

1.3 Protocol Amendment(s)

Protocol amendment 1.1 (15-Jan-2019) was integrated into previous HP002-001 protocol version (1.1 dated 23-Oct-2017). The objectives of the currently ongoing HP002-001 trial are to assess the safety and efficacy of α1H treatment in subjects with non-muscle invasive bladder cancer. The current study includes follow-up visits up to 1 month after the last administration of study treatment. Implementation of this amendment will extend the follow-up period up to 24 months post F-U 1 visit for each participating patient respectively.

Participation in the extended follow-up part of the study will be optional and done in a non-interventional way. Investigator's will follow-up patients´ health status during their routine care visits to the clinic. Participation will not require any extra visits, tests or assessments other than those planned within patients' routine care.

To participate in this extended, follow-up part of the study the subjects will need to sign a separate informed consent form to allow the study investigators to collect the data and report the data into the study.

THIS DOCUMENT CONTAINS TRADE SECRETS, AND/OR COMMERCIAL INFORMATION, DELIVERED IN CONFIDENCE AND RELIANCE THAT SUCH INFORMATION WILL NOT BE COPIED OR MADE AVAILABLE TO ANY THIRD PARTY WITHOUT THE WRITTEN CONSENT OF HAMLET PHARMA AB.

The extended follow-up period will give additional valuable information to the benefit of the study population. We will learn more about the long- term effect of the study treatment, which will be important for future patients being considered for the treatment and give additional valuable information that will help designing upcoming studies in a more optimal way.

THIS DOCUMENT CONTAINS TRADE SECRETS, AND/OR COMMERCIAL INFORMATION, DELIVERED IN CONFIDENCE AND RELIANCE THAT SUCH INFORMATION WILL NOT BE COPIED OR MADE AVAILABLE TO ANY THIRD PARTY WITHOUT THE WRITTEN CONSENT OF HAMLET PHARMA AB.

Supplementary Note 1

Table of Contents

1 Clinical Trial Protocol Statement ... 2

1.1 Approval Statement Hamlet Pharma AB ... 2

1.2 Approval Statement International Coordinating Investigator ... 2

1.3 Protocol Amendment(s) ... 2

Table of Contents ... 4

2 Trial Identification ... 8

3 Introduction and Rationale ... 8

3.1 Non-muscle Invasive Bladder Cancer ... 8

3.2 Experience with Investigational Product ... 9

3.3 Pre-clinical findings ... 9

3.3.1 Experimental tumor models ... 10

3.4 Clinical experience with HAMLET ... 11

3.5 Trial Rationale ... 12

3.5.1 Overall Trial Rationale ... 12

3.5.2 Rationale for safety assessments ... 13

3.5.3 Rationale for efficacy assessment ... 13

3.6 Ethical Consideration Statement ... 13

4 Trial Objectives and Endpoints ... 14

4.1 Objectives ... 14

4.2 Primary Endpoints ... 14

4.2.1 Adverse events (AEs); local and systemic ... 14

4.2.2 Quantification of cell shedding in urine ... 14

4.2.3 Characteristics of papillary tumors ... 15

4.3 Secondary Endpoints ... 15

4.3.1 Histopathology scoring ... 15

4.3.2 Tissue accumulation of α1H, defined by staining with specific antibodies ... 15

4.3.3 Tumor response to α1H by gene expression analysis ... 16

4.3.4 Urine cytology and apoptosis ... 16

4.3.5 Proteomic analysis of markers in urine ... 16

4.3.6 Long-term effect of the study treatment ... 16

5 Trial Design ... 16

5.1 Overall Trial Design ... 16

5.2 Sample Size ... 18

THIS DOCUMENT CONTAINS TRADE SECRETS, AND/OR COMMERCIAL INFORMATION, DELIVERED IN CONFIDENCE AND RELIANCE THAT SUCH INFORMATION WILL NOT BE COPIED OR MADE AVAILABLE TO ANY THIRD PARTY WITHOUT THE WRITTEN CONSENT OF HAMLET PHARMA AB.

5.3 Subject identification and randomisation of treatment 18
5.3.1 Subject identification 18
5.3.2 Randomisation and treatment allocation to the study subject 19
5.4 Blinding and Unblinding 19
6 Trial Population and Withdrawal 20
6.1 Subject Eligibility 20
6.2 Inclusion Criteria for All Subjects 20
6.3 Exclusion Criteria for All Subjects 21
6.4 Subject Enrolment Log 21
6.5 Subject Identification List 21
6.6 Restrictions during Trial 22
6.7 Withdrawal Criteria 22
7 Trial Schedule and Assessments 24
7.1 Schedule of Trial Procedures 24
7.2 Demographics 26
7.3 Height and Weight 26
7.4 Vital Signs 26
7.5 ECG 26
7.6 Physical Examination 27
7.7 Medical History and Concurrent Diagnosis 27
7.8 Concomitant Medication 27
7.9 Pregnancy Test 27
7.10 Adverse Events 28
7.11 Laboratory Assessments 28
7.11.1 Safety Laboratory Tests 28
7.11.2 Safety Urinalysis 29
7.11.3 Total Blood Volume 29
7.12 Cystoscopy and Photography 30
7.13 Surgery - Transurethral Resection 30
7.14 Biopsies 30
7.15 Urine Samples for Efficacy 30
7.16 End of Trial Form 31
7.17 Evaluation of long-term data 31

THIS DOCUMENT CONTAINS TRADE SECRETS, AND/OR COMMERCIAL INFORMATION, DELIVERED IN CONFIDENCE AND RELIANCE THAT SUCH INFORMATION WILL NOT BE COPIED OR MADE AVAILABLE TO ANY THIRD PARTY WITHOUT THE WRITTEN CONSENT OF HAMLET PHARMA AB.

8 Adverse Events 32
8.1 Collection of Adverse Events 32
8.2 Reporting of Adverse Events in the CRF 33
8.2.1 Actions Taken as a Consequence of an AE 33
8.3 Other Events to be Reported 33
8.3.1 Pregnancy 33
8.3.2 Overdose 34
8.3.3 Medication Error 34
8.3.4 Misuse 34
8.3.5 Abuse 34
8.3.6 Aggravation of Condition 34
8.4 Additional Reporting Requirements for Serious Adverse Events 35
8.4.1 Investigator Reporting Responsibilities 35
8.4.2 CRO Reporting Responsibilities 35
8.5 Follow-up for Final Outcome of Adverse Events 36
8.6 Assessments and Documentation of Adverse Events 36
9 Investigational Product(s) 37
9.1 Description of Investigational Product 37
9.2 Manufacturing, packaging and labelling of the Investigational Product 37
9.3 Storage and handling of the Investigational Product 37
9.4 Administration of Investigational Product 38
9.5 Non-Investigational Medicinal Products 38
9.6 Drug Accountability and Compliance Checks 38
9.6.1 Drug Accountability Investigational Product 38
9.6.2 Trial Product Destruction 38
9.7 Emergency Unblinding of Individual Subject Treatment 39
10 Statistical Methods 39
10.1 Determination of Sample Size 39
10.2 Definition of Trial Analysis Sets 39
10.3 Statistical Analysis 40
10.3.1 Disposition of Subjects 40
10.3.2 Demographics and other Baseline Characteristics 40
10.3.3 Analysis of Primary Endpoints 40
10.3.4 Analysis of Secondary Endpoints 40

THIS DOCUMENT CONTAINS TRADE SECRETS, AND/OR COMMERCIAL INFORMATION, DELIVERED IN CONFIDENCE AND RELIANCE THAT SUCH INFORMATION WILL NOT BE COPIED OR MADE AVAILABLE TO ANY THIRD PARTY WITHOUT THE WRITTEN CONSENT OF HAMLET PHARMA AB.

10.3.5 Analysis of Safety ... 41
10.3.5.1 Adverse Events ... 42
10.3.5.2 Vital Signs ... 43
10.3.5.3 Clinical Laboratory Evaluation ... 43
10.3.6 Interim Analysis ... 43
10.3.7 General Principles ... 43
11 Case Report Forms and Data Handling ... 44
11.1 Case Report Forms (CRFs) ... 44
11.2 Data Handling ... 45
11.3 Source Data ... 45
11.4 Trial Monitoring ... 46
12 Handling of an Urgent Safety Measure ... 46
13 Quality Assurance/Audit ... 47
14 Completion of Trial ... 47
14.1 Criteria for Premature Termination of the Trial and/or Trial Site ... 47
14.2 Provision for Subject Care Following Trial Completion ... 48
14.3 Archiving of Trial Documents ... 48
15 Ethics and Regulatory Authorities ... 48
15.1 Institutional Review Boards (IRBs)/Independent Ethics Committees (IECs) and Regulatory Authorities ... 48
15.2 Ethical Conduct of the Trial ... 49
15.3 Patient Information and Informed Consent ... 49
15.4 Processing of Personal Data ... 50
16 Insurance ... 50
17 Use of Information ... 50
18 Publication ... 50
19 Responsibilities ... 50
20 List of Abbreviations ... 51
21 References ... 52

THIS DOCUMENT CONTAINS TRADE SECRETS, AND/OR COMMERCIAL INFORMATION, DELIVERED IN CONFIDENCE AND RELIANCE THAT SUCH INFORMATION WILL NOT BE COPIED OR MADE AVAILABLE TO ANY THIRD PARTY WITHOUT THE WRITTEN CONSENT OF HAMLET PHARMA AB.

2 Trial Identification

This EudraCT number for this protocol is 2016-004269-14. The protocol code number is HP002-001.

3 Introduction and Rationale

3.1 Non-muscle Invasive Bladder Cancer

The prevalence of bladder cancer is about 1/4000, making it the fourth most common malignancy in the United States and the fifth in Europe. Urothelial cancer originates from the mucosal lining of the renal pelvis, the ureters and the urinary bladder. About 2000 new cases of urothelial cancer are diagnosed annually in Sweden, and the vast majority (>90%) are located in the urinary bladder [1]. The papillary non-muscle invasive tumors (T1) are removed by transurethral resection (TURB) - endoscopic removal of the tumor, but the tumors have a high recurrence rate combined with a risk for dedifferentiation. If the tumor expands into the muscular layers of the bladder i.e. "muscle-invasive uroepithelial cancer" (T2-T3), standard treatment is surgical removal of the urinary bladder (cystectomy). T4 tumors that invade the surrounding tissue require systemic therapy.

At the time of diagnosis, about 70% of uroepithelial cancers are "non-muscle invasive" [2] which means that the tumor is restricted to the bladder mucosa (TA) or the underlying lamina propria (T1). The majority of non-muscle invasive tumors grow "exophytically", and expand into the bladder lumen creating papillomatous formations. They can however also present as focal or multifocal "*cancer in situ*" (CIS), characterized by non-invasive "flat" tumors restricted to the superficial cell-layers of the uroepithelium [3, 4].

Some patients may receive topical (intravesical) treatments to prevent or delay recurrence or progression. The principle of intravesical treatment can be either chemotherapy (Mitomycin C, Epirubicin) or immunotherapy (Bacille-Calmette-Guerin - BCG). BCG treatment results in a recurrence free interval of at least 2 years in about 70% of the patients with non-muscle-invasive bladder cancers, but causes significant side effects, including granulomatous prostatitis, local discomfort and dysuria and may require systemic anti-tuberculous therapy. The incidence of life threatening complications of BCG therapy has been estimated at 0.4% [5-7], and a significant number of deaths have been reported, especially in immuno-suppressed patients. However, the problems with BCG therapy nowadays are more due to lack of efficacy than toxicity.

THIS DOCUMENT CONTAINS TRADE SECRETS, AND/OR COMMERCIAL INFORMATION, DELIVERED IN CONFIDENCE AND RELIANCE THAT SUCH INFORMATION WILL NOT BE COPIED OR MADE AVAILABLE TO ANY THIRD PARTY WITHOUT THE WRITTEN CONSENT OF HAMLET PHARMA AB.

3.2 Experience with Investigational Product

Few molecules destroy cancer cells without harming healthy tissues. While more targeted cancer therapies are starting to appear, the lack of specificity for tumor cells remains a significant problem. New concepts and innovative approaches are needed to achieve tumor specific cell death and to develop more tumor selective therapies[8, 9].

HAMLET (Human α-lactalbumin Made LEthal to Tumor cells) is a complex of partially unfolded α-lactalbumin and oleic acid that kills tumor cells and immature cells but not fully differentiated healthy cells [10]. We discovered HAMLET by serendipity, in a fraction of human milk that killed lung carcinoma cells and characterized the constituents of the complex [11]. Early *in vitro* experiments showed that HAMLET has broad anti-tumor activity with a high degree of selectivity [10]. It is encouraging that several international groups have confirmed these observations and pursue this field of research [12-18].

The tumoricidal activity of HAMLET and the relative selectivity for tumor tissue is maintained *in vivo,* as demonstrated in two human studies and several animal models. HAMLET treatment delayed the progression of human glioblastoma xenografts in nude rats and increased survival, triggering apoptotic changes in the tumor without evidence of cell death in healthy brain tissue [12]. In a placebo-controlled clinical study, topical administration of HAMLET removed skin papillomas, without side effects [19] and in patients with bladder cancer, local instillations of HAMLET killed tumor cells but not healthy cells in surrounding tissues. In addition, HAMLET triggered rapid shedding of tumor cells into the urine and caused a reduction in tumor size in patients with bladder cancer [20], with selective uptake by the tumor documented in tissue sections [21].

The investigational product, α1H, which is a further development product of HAMLET, is a synthetic peptide corresponding to the alpha1 domain of α-lactalbumin in complex with oleic acid. α1H reproduces important aspects of HAMLET's effects on tumor cells. The peptide complex is also efficient as a therapeutic agent against bladder cancer in a mouse model. There was no evidence of toxicity for healthy tissue surrounding the tumor in these mice [22].

3.3 Pre-clinical findings

HAMLET has been shown to induce apoptosis in cancer cell lines from various tissues, including urepithelial cancer cell lines [10]. Apoptosis was induced in transformed uroepithelial cancer cell lines in response to HAMLET, but were unaffected by the protein alone or the lipid, used as controls. Healthy, differentiated cells remained viable also at high concentrations of HAMLET [10].

THIS DOCUMENT CONTAINS TRADE SECRETS, AND/OR COMMERCIAL INFORMATION, DELIVERED IN CONFIDENCE AND RELIANCE THAT SUCH INFORMATION WILL NOT BE COPIED OR MADE AVAILABLE TO ANY THIRD PARTY WITHOUT THE WRITTEN CONSENT OF HAMLET PHARMA AB.

To identify conserved mechanisms of tumor cell death, we have used a combination of small hairpin RNA inhibition, proteomic and metabolomics technology (with Cold Spring Harbor and Berkeley Labs) [23]. The shRNA screen identified the c-Myc and Ras oncogenes as essential determinants of HAMLET sensitivity. Furthermore, Hexokinase 1, PFKFB1 and HIF1α modified the HAMLET sensitivity of tumor cells. Ras proteins and Hexokinase 1 were also shown to bind HAMLET in a protein array containing approximately 8000 targets and HAMLET triggered rapid metabolic paralysis in carcinoma cells, identifying classical ''Hallmarks of cancer'' as determinants of HAMLET sensitivity.

Membranes of healthy, differentiated cells remained morphologically intact, with no evidence of a change in Ras protein activity.

3.3.1 Experimental tumor models

HAMLET shows therapeutic efficacy in several animal models [12, 24, 25]. HAMLET treatment delayed the progression of human glioblastoma xenografts in nude rats and increased survival, triggering apoptotic changes in the tumor without evidence of cell death in healthy brain tissues [12]. In a placebo-controlled clinical study, topical administration of HAMLET removed skin papillomas, without side effects [19] and in patients with bladder cancer, local instillations of HAMLET killed tumor cells but not healthy cells in surrounding tissues. In patients with bladder cancer [20], HAMLET triggered rapid shedding of tumor cells into the urine and caused a reduction in tumor size was accompanied by selective uptake of HAMLET into the tumor.

The most advanced *in vitro* experiments have been performed using glioma derived tumor cell lines [26]. Experiments have been made on cell lines and using confluent gliomal cell cultures in free-floating human glioblastom (GBM) spheroids. The results demonstrate that HAMLET induces apoptosis in GBMs in cell cultures as well as tissue culture and that it can penetrate the GBM spheroids and induce apoptosis in cells throughout the entire volume of the spheroid.

In an experimental *in vivo* GBM xenograft rat model, HAMLET, and native alpha-lactalbumin, was infused in the xenograft. The overall results showed reduced tumor volume and prolonged survival of the HAMLET challenged rats as compared to the controls. Surrounding brain parenchyma did not show any histopathological signs of apoptosis and TUNEL-positive (apoptotic) cells could not be observed in the host brain [12].

In $Apc^{Min/+}$ mouse model of colon cancer, peroral HAMLET administration reduced tumor progression and mortality [21]. HAMLET accumulated specifically in tumor tissue, reduced β-catenin activity and related tumor markers. Remarkably, supplying HAMLET to the drinking

THIS DOCUMENT CONTAINS TRADE SECRETS, AND/OR COMMERCIAL INFORMATION, DELIVERED IN CONFIDENCE AND RELIANCE THAT SUCH INFORMATION WILL NOT BE COPIED OR MADE AVAILABLE TO ANY THIRD PARTY WITHOUT THE WRITTEN CONSENT OF HAMLET PHARMA AB.

water from the time of weaning also significantly prevented tumor development in these mice [21].

To evaluate if HAMLET is efficient as a topical therapeutic agent in bladder cancer, we therefore used the mouse MB49 bladder carcinoma model [24]. Rapidly growing tumors were established by intra-vesical inoculation of tumor cells and HAMLET was applied topically. Using tumor size as the therapeutic end point, a significant therapeutic effect of HAMLET was detected. A reduction in tumor development was observed in HAMLET-treated mice compared to the control group. Furthermore, by *in vivo* imaging of Alexa-Fluor labeled HAMLET, we detected accumulation in tumor tissue for at least 24 hours but not in healthy tissues surrounding the tumor. The results show that HAMLET is active as a tumoricidal agent with selective uptake in bladder tumor tissue and suggest that topical HAMLET administration should be further tested in patients with bladder cancer.

The non-toxic character of HAMLET is supported by the combined data sets from *in vitro* and *in vivo* experiments described above.

3.4 Clinical experience with HAMLET

3.4.1. Bladder cancer

In a pilot study, intra-vesical instillation of HAMLET was performed in a total of nine patients with non-muscle invasive urothelial cancer [20]. This explorative open label study focused on the impact of HAMLET on exfoliated urothelial cancer cells and if any changes could be shown on the surrounding normal uroepithelium. Four of the nine patients had newly diagnosed non-muscle invasive urothelial cancer, three had recurrence of previously diagnosed and treated disease, and two patients had CIS. All patients were handled according to clinical routines, which included an immediate waiting list for endoscopic surgery or bladder mucosal tissue sampling. While on the waiting list, the patients were asked for inclusion in the study and informed consent was given.

The patients were catheterized daily, and were given intra-vesical instillations of HAMLET (25 mg/mL, 30mL). The patients were asked to keep the HAMLET solution as long as possible (mean 2 hours, range 1-3 h), and the bladder was emptied by spontaneous voiding. Urine samples for examination of exfoliated urothelial cells and for the analysis of interleukin (IL-6 and IL-8) concentrations, were obtained prior to and after each of the instillations. At the time of transurethral resection the tumor was photographed, surgically removed, and biopsies were taken from macroscopically normal bladder tissue distant from the tumor.

THIS DOCUMENT CONTAINS TRADE SECRETS, AND/OR COMMERCIAL INFORMATION, DELIVERED IN CONFIDENCE AND RELIANCE THAT SUCH INFORMATION WILL NOT BE COPIED OR MADE AVAILABLE TO ANY THIRD PARTY WITHOUT THE WRITTEN CONSENT OF HAMLET PHARMA AB.

The repeated catheterization produced minor side effects like dysuria and local irritation, normally seen when this procedure is performed. No other systemic or local side effects were registered. Prior to treatment there were few uroepithelial cells exfoliated in urine, but after HAMLET instillation massive shedding of tumor cells was detected. Most of the shed cells showed evidence of cell death and the ratio of viable to non-viable cells was lower in the urine samples after the instillation. The papillary tumors showed macroscopic changes after HAMLET treatment in all but one of the patients with exophytic tumors. In one patient, a near complete reduction of the tumor mass had occurred. One patient with CIS showed benign histology after treatment. Histology from the exophytic tumors remaining after the treatment showed uroepithelial cancer with varying differentiation. Histology from normal bladder mucosa showed no detectable response to HAMLET.

3.4.2. Skin papillomas

Human papilloma virus (HPV) transformed keratinocytes proliferate and form warts, and most of the skin lesions remain benign [27, 28]. Cutaneous papillomas are caused by about 130 different HPV types [29]. Current treatments include cryotherapy, curettage, cautery, topical virucidal agents, laser, anti-mitotic agents and immuno-activators [30, 31]. Immunosuppressed patients run an increased risk to develop papillomas, and often carry multiple HPV types [32].

We tested HAMLET's effect as a topical therapeutic, by performing a placebo-controlled, double blind study in patients with skin papillomas [19]. The patients received topical treatment with HAMLET (0.7 mM in 0.9% NaCl) or placebo (0.9% NaCl) once a day for three weeks and the change in lesion volume was recorded. After a 3-week trial period, both the placebo and the treatment group were offered a three-week course of HAMLET in an open arm of the study. The lesion volume was reduced by ≥75% in the HAMLET group compared to 15% in the placebo group ($p<0.001$). Complete resolution of all lesions had occurred in 90% of all HAMLET treated patients after five months and the time to resolution was shorter in the patients receiving HAMLET from the start than in the placebo group. No adverse reactions were reported, and there was no difference in treatment outcome between immunocompetent and immunosuppressed patients. We concluded that topical HAMLET treatment has a beneficial and lasting effect on skin papillomas.

3.5 Trial Rationale

3.5.1 Overall Trial Rationale

The safety and efficacy of the investigational product α1H will be investigated in this trial. As a first step in development, α1H will be investigated for local treatment in patients with non-muscle invasive urothelial carcinomas. As α1H is quickly inactivated in serum by fatty acid

THIS DOCUMENT CONTAINS TRADE SECRETS, AND/OR COMMERCIAL INFORMATION, DELIVERED IN CONFIDENCE AND RELIANCE THAT SUCH INFORMATION WILL NOT BE COPIED OR MADE AVAILABLE TO ANY THIRD PARTY WITHOUT THE WRITTEN CONSENT OF HAMLET PHARMA AB.

binding proteins, we do not expect any systemic activity of this product. Serum proteins like albumin remove the lipid from the Alpha1-oleate complex, which therefore is suitable only for local administration.

3.5.2 Rationale for safety assessments

The clinical safety and tolerability of intravesical administration of α1H will be assessed by clinical examination of the subjects (see 7.4, 7.5). Clinical safety, laboratory tests will be performed and analyzed according to section 7.11.1 and urinary examination according to section 7.11.2. All adverse events reported by the subject or observed by the investigator will be recorded (section 8).

3.5.3 Rationale for efficacy assessment

This study will use intravesical instillation of α1H to investigate any apoptosis-like death in bladder cancer cells. Intravesical instillation of antitumor substances such as BCG is a well-established practice in patients with bladder cancer. Assessment of the change in tumor burden is an important feature of the clinical evaluation of cancer therapeutics. The use of tumor regression as the endpoint for phase II trials screening new agents for evidence of anti-tumor effect is supported by years of evidence suggesting that, for many solid tumors, agents which produce tumor shrinkage in a proportion of patients have a reasonable chance of subsequently demonstrating an improvement in overall survival or time to other event measures in randomized phase III studies.

3.6 Ethical Consideration Statement

Neoadjuvant intravesical treatment before TURB has been investigated for chemotherapy or thermo-chemotherapy. It was shown to be safe, with promising efficacy [20].

In the explorative HAMLET study subjects with uroepithelial cancer were subjected to repeated, intravesical instillations of HAMLET (25 mg/mL, 30 mL). The instillations were performed using intermittent urethral catheterization [20]. No systemic side effects were noted. In the same study, no effect on normal bladder mucosa was observed by histology.

In the present study of α1H instillations, we do not expect adverse effects on the normal bladder mucosa, or systemic side effects. The repeated catheterization may produce minor side effects like dysuria and local irritation, normally seen when this procedure is performed. Antibiotic prophylaxis will be used during the treatment phase.

The interval of approximately 1 month between cystoscopic diagnosis and TURB correlates fully with daily practice in the Czech Republic and represents no danger in patients with NMIBC.

Participation in this trial is voluntary and subjects are allowed to withdraw at any time.

THIS DOCUMENT CONTAINS TRADE SECRETS, AND/OR COMMERCIAL INFORMATION, DELIVERED IN CONFIDENCE AND RELIANCE THAT SUCH INFORMATION WILL NOT BE COPIED OR MADE AVAILABLE TO ANY THIRD PARTY WITHOUT THE WRITTEN CONSENT OF HAMLET PHARMA AB.

Blood samples will be drawn at scheduled visits during the trial. The expected total blood volume collected is 24 mL for each subject.

Participants in the trial will be under careful supervision by a qualified Investigator during the entire trial period. If any potentially serious reactions are observed, subjects will be withdrawn at the Investigator's discretion or according to withdrawal criteria (Section 6.7).

4 Trial Objectives and Endpoints

4.1 Objectives

The objective is to evaluate the safety of intravesical instillation of α1H in subjects with non-muscle invasive bladder cancer.

The objective is also to assess the efficacy of α1H in subjects with non-muscle invasive bladder cancer.

4.2 Primary Endpoints

4.2.1 Adverse events (AEs); local and systemic

Occurrence, intensity and relationship to the active treatment of AEs will be collected during the trial and 30 days after the last active treatment/placebo administration. Safety will also be evaluated by assessment of Vital Signs, ECG and Laboratory parameters.

4.2.2 Quantification of cell shedding in urine

Cell shedding into the urine will be used as a biomarker of the tumor response to the α1H peptide-oleate complex. α1H is expected to trigger tumor cell shedding into the urine, based on studies of HAMLET treatment, where intravesical instillations triggered tumor cell shedding in subjects with bladder cancer. In addition, a significant increase in dead epithelial cells was observed after HAMLET treatment.

- Cell shedding will be quantified by counting the total number of epithelial cells in a unit of urine (cell type and number per mL). The change in cell shedding will be quantified in each individual, by comparing cell numbers in samples obtained before and after each instillation (intra-individual comparison). This will be repeated at each instillation (intra-individual comparisons and group-wise, by comparing subjects receiving active drug versus placebo.

THIS DOCUMENT CONTAINS TRADE SECRETS, AND/OR COMMERCIAL INFORMATION, DELIVERED IN CONFIDENCE AND RELIANCE THAT SUCH INFORMATION WILL NOT BE COPIED OR MADE AVAILABLE TO ANY THIRD PARTY WITHOUT THE WRITTEN CONSENT OF HAMLET PHARMA AB.

- Ratios of live to dead cells will be determined in a unit of urine. This will be performed either using a Burker chamber or an automated cell counting machine.

- Malignant cells will be identified by the pathologist, using standard cytology techniques.

4.2.3 Characteristics of papillary tumors

The response of the papillary tumors to α1H will be characterized by *in vivo* imaging during examination by cystoscopy. The resected tumor will be characterized by histopathology. The α1H peptide-oleate complex is expected to trigger a reduction in tumor size and a change in shape, based on studies of HAMLET in patients with bladder cancer and results from the murine bladder cancer model.

- The number of papillary tumors will be quantified by a visual survey of the bladder mucosa at the onset of treatment and after the last instillation of α1H.

- A change in papillary tumor shape and size will be documented by photography, video recording and specific measurements will be made by analytical software.

4.3 Secondary Endpoints

4.3.1 Histopathology scoring

- Tissue changes will be quantified by histopathology, using established parameters for scoring of Grade and Stage/Invasiveness. Biopsies will be analysed by a designated study pathologist.

- Changes in tumor tissue will be characterized by immunohistochemistry for tumor markers, cell death, proliferation, and inflammation.

4.3.2 Tissue accumulation of α1H, defined by staining with specific antibodies

- Tumor-specific uptake of α1H by tumor tissue will be compared to healthy tissue in individual hosts. Tissue sections will be stained with specific antibodies.
- Uptake of α1H by tumor tissue will be quantified by staining of tissue sections with specific antibodies. Uptake will be compared to healthy tissue in individual hosts.

THIS DOCUMENT CONTAINS TRADE SECRETS, AND/OR COMMERCIAL INFORMATION, DELIVERED IN CONFIDENCE AND RELIANCE THAT SUCH INFORMATION WILL NOT BE COPIED OR MADE AVAILABLE TO ANY THIRD PARTY WITHOUT THE WRITTEN CONSENT OF HAMLET PHARMA AB.

4.3.3 Tumor response to α1H by gene expression analysis

- Changes in the cancer-associated pathways will be analysed in tumor biopsies and shed cells by whole genome transcriptional profiling. Microarray technology will be used for this purpose. Changes in cancer-associated signalling pathways will be compared between the treatment and placebo groups.

4.3.4 Urine cytology and apoptosis

- Cells in urine will be examined before and after instillation, using standard cytology techniques and parameters defining tumor grade and stage.

- Apoptotic changes in the shed cells will be quantified, as well as other markers of cell death.

4.3.5 Proteomic analysis of markers in urine

- Changes in protein markers will be quantified by comparing samples obtained before and after the instillation of α1H.

4.3.6 Long-term effect of the study treatment

- Characterization of progression or non-progression of the study disease during the extended follow-up period of 24 months.
- Time to first recurrence i.e. time between TURB (Visit 7) to next TURB, within the extended follow-up period of 24 months.

5 Trial Design

5.1 Overall Trial Design

Figure 1: Course of the main trial

THIS DOCUMENT CONTAINS TRADE SECRETS, AND/OR COMMERCIAL INFORMATION, DELIVERED IN CONFIDENCE AND RELIANCE THAT SUCH INFORMATION WILL NOT BE COPIED OR MADE AVAILABLE TO ANY THIRD PARTY WITHOUT THE WRITTEN CONSENT OF HAMLET PHARMA AB.

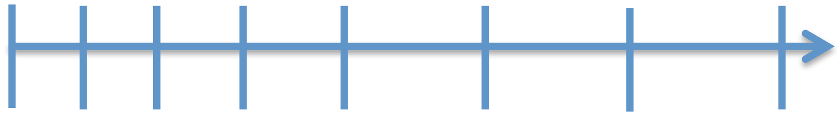

Day	1	3	5	8	15	22	30	52
Visit	**1**	**2**	**3**	**4**	**5**	**6**	**7**	**F-U**
α1H or Placebo	◆	◆	◆	◆	◆	◆		
Urine sample	◆	◆	◆	◆	◆	◆	◆	
Cystoscopy	◆						◆	
Surgery (TURB)							◆	
Safety follow-up								◆

This will be a single centre, placebo controlled, double blind trial in subjects from 18 years of age with non-muscle invasive bladder cancer awaiting transurethral surgery. The subjects will receive intravesical instillation of either α1H or placebo on 6 occasions during a period of 22 days. The study will be divided in two periods; the main study period and an optional follow-up period. The duration of the main study including the safety follow-up visit will be about 52 days.

The duration of the optional follow-up period will be 24 months. This part of the study is planned to obtain long-term efficacy data. Investigators will, after FU visit 1, assess patients' health status from routine care visits and report a summary of the observations every 6 months. A separate form will be used for reporting. For the patients accepting to participate, the total study duration will be about 52 days plus 24 months.

<u>Day 0</u>

The subject will be considered for enrolment in the study, based on a diagnosis of bladder cancer. The investigator will explain the nature, purpose and risks of the trial and provide the subject with a copy of the Patient Information and Informed Consent (Appendix 8). The subjects will be given the informed consent form and will be asked to consider and evaluate participation in the study in accordance with the Helsinki Declaration and the local applicable laws.

<u>Visits 1-6, Treatment Period</u>

At visit 1, subjects will sign and date the Patient Information and Informed Consent (Appendix 8) before entering the trial. The subject will be provided with a copy of the consent form and the signed consent will be documented by the investigator in the Case Report Form (CRF) and subject's medical record.

THIS DOCUMENT CONTAINS TRADE SECRETS, AND/OR COMMERCIAL INFORMATION, DELIVERED IN CONFIDENCE AND RELIANCE THAT SUCH INFORMATION WILL NOT BE COPIED OR MADE AVAILABLE TO ANY THIRD PARTY WITHOUT THE WRITTEN CONSENT OF HAMLET PHARMA AB.

Should there be any amendments to the Final Protocol, such that would directly affect the subject's participation in the trial e.g. a change in any procedure, the Patient Information and Informed Consent document must be amended to incorporate this modification and the subject must agree to sign this amended form indicating that they re-consent to participating in the trial.

At scheduled visits 1-6, on days 1, 3, 5, 8, 15 and 22, subjects will be treated. The timing of each visit is defined by a time frame as indicated in the study chart below. Visits outside this time frame should be recorded by the investigator in the subject's medical record as well as in the CRF.

Visit 7

At Visit 7, the bladder tumors will be characterized prior to scheduled surgery. Subjects will undergo similar procedures and analyses as for visit 1. Remaining tumors will be removed by TURB (according to EAU Guidelines recommendations) and tissues will be obtained for analyses.

Follow-up Visit 1

F-U 1 Visit will take place 30 days after the last administration of study treatment.

Long-term Follow-up Visits (optional)

Long-term efficacy data will be collected from patient's routine care visits every 6 months.

5.2 Sample Size

The sample size of 20 subjects on active treatment and 20 subjects on placebo is based on results from the previous study with HAMLET [19].

5.3 Subject identification and randomisation of treatment

All enrolled subjects will be randomized 1:1 to two study treatment groups at Visit 1.

5.3.1 Subject identification

Subject screening numbers will be generated sequentially to subjects consenting to participate in the study.

Subject Randomization number will be assigned sequentially to each randomized subject and used throughout the study.

THIS DOCUMENT CONTAINS TRADE SECRETS, AND/OR COMMERCIAL INFORMATION, DELIVERED IN CONFIDENCE AND RELIANCE THAT SUCH INFORMATION WILL NOT BE COPIED OR MADE AVAILABLE TO ANY THIRD PARTY WITHOUT THE WRITTEN CONSENT OF HAMLET PHARMA AB.

5.3.2 Randomisation and treatment allocation to the study subject

A unique vial code will be allocated and printed on the label of each vial containing the study investigational product (α1H or placebo). Vial code will consist of the subject randomisation number and Visit number (number 1 - 6) indicating IMP administration sequence. **The randomisation plan** will link each vial and subject with either placebo or active treatment arm. The plan will be generated centrally by NEOX unblinded biostatistician using IBM SPSS Statistics for Windows, Version 23.0. (Armonk, NY: IBM Corp.) and will be available only to unblinded study members.

NEOX study biostatistician will generate a **Study treatment allocation list,** using IBM SPSS Statistics for Windows, Version 23.0. (Armonk, NY: IBM Corp.). This list links each study subject randomisation number (in consecutive numeric row) with unique 6 vial codes and will be available to all study members through eCRF system and on paper list.

At the randomisation visit, each subject who meets all eligibility criteria will be given the lowest randomisation number available at the site. Based on the allocated randomisation number and Study treatment allocation list, 6 vials with investigational product will be allocated to each randomised subject. First vial marked Visit 1 of 6 allocated vials will be administered during the randomisation visit, remaining 5 vials (Visits 2-6) will be administered during the consecutive study treatment visits as specified in the protocol. Investigational product administration will be documented with date, time and vial code in the source documentation and eCRF.

5.4 Blinding and Unblinding

Every effort was taken to blind the investigational product. Therefore, the vials are labelled such that no difference can be detected in the outer appearance.

Study medication will be supplied frozen in 50-mL glass vials. Both active substance and placebo will be of similar appearance. Therefore, normal glass vials and normal dosing syringes will be used without any risk to the blinding procedure.

Data will be collected in an observer-blind manner. By observer-blind, it is meant that during the course of the study, the active treatment / placebo recipient and those responsible for the evaluation of any study endpoint (e.g. safety, biomarkers, and efficacy variables) will all be unaware what study treatment (active treatment / placebo) was administered.

The laboratory in charge of the sample testing will be blinded to the treatment, and codes will be used to link the subject and study (without any link to the treatment assigned to the subject) to each sample.

THIS DOCUMENT CONTAINS TRADE SECRETS, AND/OR COMMERCIAL INFORMATION, DELIVERED IN CONFIDENCE AND RELIANCE THAT SUCH INFORMATION WILL NOT BE COPIED OR MADE AVAILABLE TO ANY THIRD PARTY WITHOUT THE WRITTEN CONSENT OF HAMLET PHARMA AB.

The study site will be provided with a set of sealed **Unblinding Envelopes**. Each envelope will be marked by the subject's Randomisation number, study identification and the unblinding instructions. Each envelope will contain information on the content of the subject allocated vials (placebo or active substance).

Unblinding Envelopes will be kept at the clinic (in an access-restricted repository) to enable the investigator to break the code for safety purposes, if required during the study. If it becomes necessary to break the code during the study, the date, time and reason will be recorded in the subject's source data, on the individual envelope and in eCRF. Every attempt should be made to contact the sponsor before un-blinding a subject. The sponsor or CRO NEOX must be contacted immediately to decide if the subject should be withdrawn from the study.

At the time of the interim analysis after end of the main study, the study will be unblinded and the results evaluated. Refer to Section 10 Statistical Methods for more details regarding the interim analyses to be performed. Patient's participating in the extended Long-term follow-up period will be included and followed unblinded for 24 months after F-U 1 visit..

6 Trial Population and Withdrawal

6.1 Subject Eligibility

The (sub)investigator should only enrol subjects who meet all eligibility criteria, are not put at undue risk by participating in the trial and can be expected to comply with the protocol.

The subject's eligibility for the clinical trial must be checked according to the inclusion and exclusion criteria at visits specified in "Schedule of Trial Procedures" (see section 7.1).

Any implementation of national requirements/law for the subject's participation in the clinical trial must be ensured and described in the submission documentation to authorities/ethics committees, as applicable.

6.2 Inclusion Criteria for All Subjects

1. Signed and dated informed consent.

2. Patient with non-muscle invasive papillary bladder cancer (NMIBC) based on cystoscopy appearance, on the waiting list for TURB.

3. Male and female subjects, 18 years or older.

4. Negative pregnancy test in women of childbearing potential.

THIS DOCUMENT CONTAINS TRADE SECRETS, AND/OR COMMERCIAL INFORMATION, DELIVERED IN CONFIDENCE AND RELIANCE THAT SUCH INFORMATION WILL NOT BE COPIED OR MADE AVAILABLE TO ANY THIRD PARTY WITHOUT THE WRITTEN CONSENT OF HAMLET PHARMA AB.

5. Appropriate methods of contraception in women of childbearing potential during study.

6. Patients should be able to keep the content of the bladder for at least one hour.

6.3 Exclusion Criteria for All Subjects

1. Patient with a previous history of muscle invasive bladder cancer.

2. Patient with a history of NMIBC with an interval shorter than 6 months after previous TURB.

3. Previous intravesical BCG immunotherapy in the last 12 months.

4. Previous intravesical chemotherapy in the last 12 months.

5. Participants with any other cancer diagnosis within the last 5 years (except of skin basaliomas).

6. Acute urinary tract infection

7. Participants with prior radiotherapy or systemic chemotherapy.

8. Participants receiving any other investigational agent or non-marketed product one month prior to Visit 1 and during the trial.

9. Any concurrent illness that may render a participant ineligible or limit compliance with study requirements.

10. Previously enrolled in this trial.

6.4 Subject Enrolment Log

All subjects screened for this study will be logged on a screening log. This log will list whether subjects were enrolled or not. If a subject is not enrolled, the main reason will be listed. The list will be prepared according to local regulations about personal data protection.

6.5 Subject Identification List

The investigator must maintain a list of all subjects randomised/treatment assigned at the trial site including each subject's identity, date of enrolment and corresponding subject ID so that

THIS DOCUMENT CONTAINS TRADE SECRETS, AND/OR COMMERCIAL INFORMATION, DELIVERED IN CONFIDENCE AND RELIANCE THAT SUCH INFORMATION WILL NOT BE COPIED OR MADE AVAILABLE TO ANY THIRD PARTY WITHOUT THE WRITTEN CONSENT OF HAMLET PHARMA AB.

any subject may be identified if required for any reason. The list is kept by the investigator and must not be copied or retained by Hamlet Pharma or CRO.

At Visit 1 each subject must be assigned a unique subject ID to protect the subject's identity and which will be used in lieu of the subject's name when the investigator reports trial-related data.

6.6 Restrictions during Trial

The use of drugs, defined as disallowed in the exclusion criteria, is not permitted during the trial and as also defined in section 6.7.

Use of concomitant treatment must be recorded in the subject’s medical record and the Case Report Form (CRF) (treatment/drug name, dose, indication and dates of start and stop).

6.7 Withdrawal Criteria

Subjects **may** withdraw from the trial for any of the following reasons:

1. *Unacceptable treatment efficacy:* the investigator is free to withdraw the subject at any time based on a medical judgement.
2. *Unacceptable adverse events:* any adverse event that the investigator or the subject considers unacceptable.
3. *Exclusion criteria:* any exclusion criteria which emerge/become apparent during the subject’s participation in the clinical trial.
4. *Voluntary withdrawal:* subjects are free to withdraw from the clinical trial at any time and for any reason. If applicable, the subject’s legal representative can withdraw the subject from the trial.
5. *Other reasons:* other reasons than stated above which require the subject to (be) withdraw(n) should be specified.

Subjects MUST be withdrawn if they are found to have become pregnant or experience an allergic reaction to α1H.

Based on the safety profile of α1H, there are no expected risks that would require specific withdrawal criteria in addition to the reasons described above.

Reason(s) for withdrawal must be recorded in the CRF and medical records.

THIS DOCUMENT CONTAINS TRADE SECRETS, AND/OR COMMERCIAL INFORMATION, DELIVERED IN CONFIDENCE AND RELIANCE THAT SUCH INFORMATION WILL NOT BE COPIED OR MADE AVAILABLE TO ANY THIRD PARTY WITHOUT THE WRITTEN CONSENT OF HAMLET PHARMA AB.

Subjects who are discovered, after enrolment/randomisation, not to have fulfilled all in-/exclusion criteria at the time of enrolment should discontinue treatment unless the investigator, based on clinical and ethical evaluation, finds discontinuation inappropriate.

Any subject prematurely withdrawn from the study will attend Visit 7 according to the protocol and FU visit 30 ±5 days from the last study treatment administration. For subjects withdrawn from treatment or trial, AEs should be followed up as described in section 8.5.

THIS DOCUMENT CONTAINS TRADE SECRETS, AND/OR COMMERCIAL INFORMATION, DELIVERED IN CONFIDENCE AND RELIANCE THAT SUCH INFORMATION WILL NOT BE COPIED OR MADE AVAILABLE TO ANY THIRD PARTY WITHOUT THE WRITTEN CONSENT OF HAMLET PHARMA AB.

7 Trial Schedule and Assessments

7.1 Schedule of Trial Procedures

Study chart for the main study

	**Visit 0**	**Visit 1**	**Visits 2 & 3**	**Visits 4, 5 & 6**	**Visit 7**	**F-U1 8**
**Day/Month**	Day -28 to -1	Day 1	Day 3 & 5	Day 8, 15 & 22	Day 30	Day 52
**Visit window**			±1 Day, each visit	±2 Days, each visit	±5 Days	±5 Days
Treatment / Intravesical instillation at hospital		X	X	X		
Informed consent	(X)	X				
Subject demographics		X				
In-/exclusion criteria		X				
Pregnancy test		X			X	
Medical history and concurrent diagnosis.		X				
Concomitant medication		X	X	X	X	X
Physical examination		X			X	
Height and weight		X				
Randomisation		X				
Adverse Event(s)		X	X	X	X	X
Vital signs (BP, pulse)		X			X	X
ECG		X			X	
Blood sample (haematology and biochemistry)		X			X	X
Urinalysis (safety)		X			X	X
Urinalysis (efficacy)		X	X	X		
Cystoscopy + Photo		X			X	

THIS DOCUMENT CONTAINS TRADE SECRETS, AND/OR COMMERCIAL INFORMATION, DELIVERED IN CONFIDENCE AND RELIANCE THAT SUCH INFORMATION WILL NOT BE COPIED OR MADE AVAILABLE TO ANY THIRD PARTY WITHOUT THE WRITTEN CONSENT OF HAMLET PHARMA AB.

	Visit 0	Visit 1	Visits 2 & 3	Visits 4, 5 & 6	Visit 7	F-U1 8
**Day/Month**	Day -28 to -1	Day 1	Day 3 & 5	Day 8, 15 & 22	Day 30	Day 52
**Visit window**			±1 Day, each visit	±2 Days, each visit	±5 Days	±5 Days
Trans Urethral Resection and Biopsy (TURB) i.e. surgery of any remaining tumors.					X	
Collection of long-term data from patients' medical records (optional)						

30 days after the last IMP administration, Visit F-U1 will take place. Investigator will assess occurrence of any on-going or new AEs. AEs will be followed until final outcome as described in Section 8.5

After F-U 1 visit, provided the patients sign a separate Informed Consent Form, the patient will be enrolled in an optional non-interventional Long-term Follow-Up period. Investigators will assess patients' health status from routine care visits and report a summary conclusion of the long-term effect of study treatment every 6 months. AEs will not be collected during the Long-term follow up of the study. A separate form will be used for reporting. For the patients accepting to participate in this part of the study the total study duration will be about 52 days plus 24 months.

THIS DOCUMENT CONTAINS TRADE SECRETS, AND/OR COMMERCIAL INFORMATION, DELIVERED IN CONFIDENCE AND RELIANCE THAT SUCH INFORMATION WILL NOT BE COPIED OR MADE AVAILABLE TO ANY THIRD PARTY WITHOUT THE WRITTEN CONSENT OF HAMLET PHARMA AB.

7.2 Demographics

Demographic data will comprise of:
Date of birth
Sex
Ethnic origin
Race

The subjects will self-report their ethnicity (Hispanic or Latino, not Hispanic or Latino) and race (American Indian or Alaska Native, Asian, Black or African American, Native Hawaiian or other Pacific Islander, White, Other).

7.3 Height and Weight

The subject's height must be measured (without shoes) and weight must be determined (in indoor clothing and without shoes).

7.4 Vital Signs

Vital signs (resting blood pressure and heart rate) must be assessed.

Recording of vital signs comprises:

- Heart rate (beats per minute), supine after 5 min rest
- Blood pressure, systolic and diastolic (mmHg), supine after 5 min rest

The same arm will be used for all measurements. The arm (right or left) used for measurement will be recorded in the CRF.

Assessment of vital signs resulting in abnormal values will be repeated in order to exclude an erroneous assessment. Individual results will be classified as "normal", "abnormal with no clinical significance" or "abnormal with clinical significance".

7.5 ECG

A standard 12-lead ECG will be recorded after 5 minutes of rest in the supine position. Each recording shall clearly be marked with the Subject number, date and time of the recording. The following ECG parameters will be recorded: heart rate, PR interval, QRS duration, QT interval, QTc interval. The ECG will be performed at Visit 1 and Visit 7. Patients with QTcB interval > 450 ms for males and 470 ms for females or other relevant pathological changes in the ECG in opinion of the investigator should be excluded. These intervals apply at screening. Clinically

THIS DOCUMENT CONTAINS TRADE SECRETS, AND/OR COMMERCIAL INFORMATION, DELIVERED IN CONFIDENCE AND RELIANCE THAT SUCH INFORMATION WILL NOT BE COPIED OR MADE AVAILABLE TO ANY THIRD PARTY WITHOUT THE WRITTEN CONSENT OF HAMLET PHARMA AB.

significant ECG findings from the screening visit should be recorded as medical history. Clinically significant ECG findings from subsequent visits should be recorded as an AE.

7.6 Physical Examination

A physical examination including general appearance, regional lymph nodes and dermatologic examination of the skin in general must be performed.

7.7 Medical History and Concurrent Diagnosis

Medical history must be recorded:

- Bladder cancer: Date when bladder cancer was diagnosed must be recoded.
- Other medical history within the last 12 months

7.8 Concomitant Medication

At each study visit/contact, the investigator should question the subject about any medication taken by the subject.

All concomitant medication, with the exception of vitamins and/or dietary supplements, administered during the time period from randomisation to last administration of study drug, are to be recorded in the eCRF. This also applies to concomitant medication administered prophylactically in anticipation of reaction to the active treatment and any medication intended to treat an AE.

A prophylactic medication is a medication administered in the absence of ANY symptom and in anticipation of a reaction to the active treatment (e.g. an anti-pyretic, corticosteroids, etc.).

Similarly, concomitant medication administered for the treatment of a SAE at any time, must be recorded on the SAE Report / SAE screens in the eCRF, as applicable.

Use of non-marketed/other investigational products one month prior to Visit 1 and during the trial is not permitted.

Refer to Appendix 2 for the definition of a SAE and to Section 8.6 for the applicable reporting periods.

7.9 Pregnancy Test

A urine pregnancy test must be performed at the trial site at Visit 1 and Visit 8 in female subjects of childbearing potential. Hamlet Pharma will provide the test kits.

THIS DOCUMENT CONTAINS TRADE SECRETS, AND/OR COMMERCIAL INFORMATION, DELIVERED IN CONFIDENCE AND RELIANCE THAT SUCH INFORMATION WILL NOT BE COPIED OR MADE AVAILABLE TO ANY THIRD PARTY WITHOUT THE WRITTEN CONSENT OF HAMLET PHARMA AB.

7.10 Adverse Events

Adverse events must be assessed and recorded as specified in section 8.

7.11 Laboratory Assessments

7.11.1 Safety Laboratory Tests

Samples for analysis of parameters must be taken according to the schedule of trial procedures (section 7.1) or on withdrawal from or early completion of the treatment phase of the clinical trial.

The following analyses will be performed on the blood samples: Analyses	Parameter
Haematology	Haemoglobin<br>Haematocrit<br>Red blood cell (RBC) count<br>Mean corpuscular volume (MCV)<br>White blood cell (WBC) count, including differential count<br>Platelet count
Biochemistry (serum/plasma)	Urea<br>Creatinine<br>Albumin<br>Sodium<br>Potassium<br>Chloride<br>Calcium<br>Phosphate<br>Alkaline phosphatase (ALP)<br>Glucose

If any laboratory results are abnormal, the investigator should follow-up the subject as clinically appropriate.

Handling and shipment instructions are provided in laboratory manuals by the local laboratory at the Motol University Hospital.

THIS DOCUMENT CONTAINS TRADE SECRETS, AND/OR COMMERCIAL INFORMATION, DELIVERED IN CONFIDENCE AND RELIANCE THAT SUCH INFORMATION WILL NOT BE COPIED OR MADE AVAILABLE TO ANY THIRD PARTY WITHOUT THE WRITTEN CONSENT OF HAMLET PHARMA AB.

7.11.2 Safety Urinalysis

Samples for analysis of the parameters listed below must be taken as specified in the schedule of trial procedures (section 7.1) or on withdrawal from or early completion of the treatment phase of the clinical trial.

Analyses	Parameter
Urine analysis	pH<br>Calcium<br>Phosphate<br>Creatinine<br>Urea<br>Sediment<br>Glucose<br>Ketones<br>Nitrate<br>Proteins<br>Cytology

7.11.3 Total Blood Volume

The following blood volumes will be drawn per subject:

Sample type	Volume per sample (mL)	Number of samples Visit 1	Number of samples Visit 7	Number of samples F-U	Total (mL)
Haematology	2	1	1	1	6
Biochemistry	4	1	1	1	12
Glycemia	2	1	1	1	6
Total volume		8	8	8	24

THIS DOCUMENT CONTAINS TRADE SECRETS, AND/OR COMMERCIAL INFORMATION, DELIVERED IN CONFIDENCE AND RELIANCE THAT SUCH INFORMATION WILL NOT BE COPIED OR MADE AVAILABLE TO ANY THIRD PARTY WITHOUT THE WRITTEN CONSENT OF HAMLET PHARMA AB.

7.12 Cystoscopy and Photography

Based on patient's medical record (Visit 0) where patients have been subjected to cystoscopy using a flexible cysto-nephro fiberscope to document the diagnosis of non-muscle invasive bladder cancer, patient may be eligible for the study. The cystoscopy should follow standard clinical routines regarding use of antibiotics, premedication, local anesthesia and disinfectants. Any drug given per orally or parenterally should be documented in the patient file. At cystoscopy, tumor size will be assessed and lesions documented using endoluminal photography. The tumors will be identified, documented and photographed. An endoluminal grasp forceps should be used to enable estimation of the size of the tumors, and should be visible on at least one high quality photography. The same tumors should if possible be identified at the last visit (Visit 7) and again be documented repeating the same use of standardized technique for photography and size estimation as in the first cystoscopy. The assessment of an individual patient must be carried out by the same investigator at Visit 1 and Visit 7 to ensure highest possible repeatability.

Flow chart and Documentation of lesions using cystoscopy is given in Appendix 4.

7.13 Surgery - Transurethral Resection

At visit 7, subjects will be investigated by cystoscopy using the flexible cysto-nephro fiberscope. If any remaining tumors can be identified these will be removed by immediate TURB. The investigator having done the cystoscopy at Visit 1 should preferably do the surgery at visit 7.

7.14 Biopsies

At surgery (Visit 7), tumor biopsies will be collected, fixed in 4% fresh PFA in PBS for 24h. Fixed samples will then be embedded in paraffin and sectioned for further analysis.

7.15 Urine Samples for Efficacy

Prior to and after each instillation of α1H (Visit 1 – Visit 6), urine samples will be obtained and analyzed. Cells in un-centrifuged urine will be counted by light microscopy using a Bürker chamber or automated counter. Viability will be determined by trypan blue exclusion.

For urine cytology, cells concentrated from 10-30 mL urine will be used. Urine will be centrifuged (Cytospin 3, Shandon, Cheshire, GB) at 500 rpm for 5 minutes onto L-lysine coated microscope slides, fixed and stored at –20°C. The cell morphology will be determined after staining with hematoxylin and eosin according to standard procedure.

THIS DOCUMENT CONTAINS TRADE SECRETS, AND/OR COMMERCIAL INFORMATION, DELIVERED IN CONFIDENCE AND RELIANCE THAT SUCH INFORMATION WILL NOT BE COPIED OR MADE AVAILABLE TO ANY THIRD PARTY WITHOUT THE WRITTEN CONSENT OF HAMLET PHARMA AB.

Apoptotic cells will be identified by the TUNEL assay (Roche, Basel, Switzerland). Briefly, the slides will be thawed at room temperature, washed twice in PBS and permeabilized with sodium citrate (0.1%) and Triton X-100 (0.1%), exposed to the TUNEL reaction mixture, incubated in a humidified chamber at 37°C in 5% CO_2 for 1h, cover-slipped with mounting medium (Vectashield, Vector Labs Inc., Burlingame, CA, USA) and examined by fluorescence microscopy using the LSM META 510 software package (Carl Zeiss, Jena, Germany). Representative cells will be photographed using a CCD camera (Diagnostic Instruments Inc., Michigan, USA.).

In addition, urine samples will be frozen at -80° C and examined at the Department of Pathology at Lund University Hospital.

7.16 End of Trial Form

The End of Trial Form must be completed for all subjects who have signed informed consent for the main study. This includes e.g. date of last instillation, last attended scheduled visit number, primary reason for withdrawal, etc.

7.17 Evaluation of long-term data

In addition to the mandatory F-U 1 visit at 30 days after the last dose of study treatment, long-term follow-up is planned. Long-term follow-up of the patients is optional and non-interventional by nature. Participation does not require extra visits other than those planned within patient's routine care. Additional tests or assessments will not take place.

To take part in this extended follow up period, patients will need to sign an informed consent form that allow the study site to retrospectively (some patients have already finished the study) and prospectively collect information about patient's health status during their routine care visits. Patients being referred to the study site from other clinics will not need to visit the study site. Study investigators will contact their physician and collect follow-up information over the phone.

Investigators will report a summary conclusion of patient's health status every 6 months. A separate form will be used for reporting. For the patients accepting to participate in this part of the study the total study duration will be about 52 days plus 24 months.

THIS DOCUMENT CONTAINS TRADE SECRETS, AND/OR COMMERCIAL INFORMATION, DELIVERED IN CONFIDENCE AND RELIANCE THAT SUCH INFORMATION WILL NOT BE COPIED OR MADE AVAILABLE TO ANY THIRD PARTY WITHOUT THE WRITTEN CONSENT OF HAMLET PHARMA AB.

8 Adverse Events

- Adverse events and serious adverse events are defined in Appendix 2: Definitions of Adverse Events and Serious Adverse Events.
- Classification of adverse events in terms of severity, causality and outcome are defined in Appendix 3: Classification of Adverse Events.

8.1 Collection of Adverse Events

Adverse events must be collected from the signing of the informed consent form until the End of the main trial (F-U 1 Visit , Day 52). Investigator will assess occurrence of any on-going or new AEs. AEs will be followed until final outcome. AEs will not be collected during the Long-term follow up of the study.

AEs must be assessed by medically qualified personnel.

At all visits, the subject will be asked a non-leading question by the investigator: "How have you felt since I saw you last?" No specific symptoms should be asked for. It is important that the investigator also observes the subject for any changes not reported by the subject and records these changes.

If there are no AEs to record, no further questions should be asked and "NO" should be stated in the CRF. In case there are one or more AEs to record, "YES" should be stated.

All diagnoses, symptom(s), sign(s) or finding(s) with a start date **after first dose of study drug** must be recorded as (S)AEs, except those related to study procedure. The latter have to be recorded as (S)AEs **after informed consent** has been obtained.

A surgical procedure that was planned prior to administration of study drug by any physician treating the subject should not be recorded as an AE (however, the condition for which the surgery is required may be an AE).

In the following differentiation between medical history and AEs, the term '*condition*' may include abnormal physical examination findings, symptoms, diseases, laboratory or ECG findings:

- Conditions that were present before first dose of study drug and for which no symptoms or treatment are present until administration of study drug are recorded as <u>medical history</u> (e.g. seasonal allergy without acute complaints)
- Conditions that started before first dose of study drug and for which symptoms or treatment are present after first dose of study drug, at *unchanged intensity*, are recorded as <u>medical history</u> (e.g. allergic pollinosis)

THIS DOCUMENT CONTAINS TRADE SECRETS, AND/OR COMMERCIAL INFORMATION, DELIVERED IN CONFIDENCE AND RELIANCE THAT SUCH INFORMATION WILL NOT BE COPIED OR MADE AVAILABLE TO ANY THIRD PARTY WITHOUT THE WRITTEN CONSENT OF HAMLET PHARMA AB.

- Conditions that started or deteriorated after first dose of study drug will be documented as (S)AEs
- Conditions assessed as **related to study** that start or deteriorate **after** signed informed consent must be documented as (S)AEs.

8.2 Reporting of Adverse Events in the CRF

Adverse events reported by the subject or observed by the investigator must be recorded on the adverse event form of the CRF and should be described in the following manner:

The *AE term* will be in precise English medical terminology (i.e. not necessarily the exact words used by the subject). Whenever possible, a specific diagnosis should be stated.

The *duration* of the AE must be reported as the start date and stop date of the event. In addition, it must be recorded whether the AE started prior to start of trial medication.

AEs must be classified in terms of severity, causality and outcome according to the definitions in Appendix 3: Classification of Adverse Events.

8.2.1 Actions Taken as a Consequence of an AE

Action taken with trial treatment: Any action taken with trial medication as a consequence of the AE must be recorded (dose not changed, dose reduced, dose increased, drug interrupted, drug withdrawn, not applicable, unknown).

Other action taken: Any other action taken as a result of the AE must be recorded (none, concomitant medication, concurrent procedure).

Withdrawn due to AE: It must be recorded whether the AE leads to withdrawal from the trial.

8.3 Other Events to be Reported

8.3.1 Pregnancy

Any pregnancy occurring during the clinical trial must be reported to NEOX within 24 hours of first knowledge using the (paper) Pregnancy Follow Up Form (Part I). All such pregnancies must be followed up until delivery or termination and final outcome must be reported on the (paper) Pregnancy Follow Up Form (Part II) within 24 hours of first knowledge.

The completed Pregnancy Follow Up Forms must be faxed or scanned or e-mailed to NEOX.

Please also confer with section 6.7, Withdrawal Criteria.

THIS DOCUMENT CONTAINS TRADE SECRETS, AND/OR COMMERCIAL INFORMATION, DELIVERED IN CONFIDENCE AND RELIANCE THAT SUCH INFORMATION WILL NOT BE COPIED OR MADE AVAILABLE TO ANY THIRD PARTY WITHOUT THE WRITTEN CONSENT OF HAMLET PHARMA AB.

8.3.2 Overdose

Overdose refers to the administration of a quantity of a medicinal product given per administration or per day, which is above the protocol-defined dosage.

The term overdose must be documented on the adverse event form of the CRF book. In addition, AEs originating from overdose must be documented on a separate line.

8.3.3 Medication Error

Medication error refers to any unintentional error in the dispensing or administration of a medicinal product while in the control of the investigator or subject. Broadly, medication errors fall into four categories: wrong medication, wrong dose (including strength, form, concentration, amount), wrong route of administration or wrong subject.

The medication error must be documented on the adverse event form of the CRF book. In addition, AEs originating from a medication error must be documented on a separate line specifying the category of error (see definitions above).

8.3.4 Misuse

Misuse refers to situations where the medicinal product is intentionally and inappropriately used not in accordance with the protocol.

The term misuse must be documented on the adverse event form of the CRF book. In addition AEs originating from misuse must be documented on a separate line.

8.3.5 Abuse

Abuse relates to the sporadic or persistent, intentional excessive use of an investigational product which is accompanied by harmful physical or psychological effects.

The term abuse must be documented on the adverse event form of the CRF book. In addition, AEs originating from abuse must be documented on a separate line.

8.3.6 Aggravation of Condition

Any clinically significant aggravation/exacerbation/worsening of any medical condition(s), compared to baseline, must be reported as an AE.

THIS DOCUMENT CONTAINS TRADE SECRETS, AND/OR COMMERCIAL INFORMATION, DELIVERED IN CONFIDENCE AND RELIANCE THAT SUCH INFORMATION WILL NOT BE COPIED OR MADE AVAILABLE TO ANY THIRD PARTY WITHOUT THE WRITTEN CONSENT OF HAMLET PHARMA AB.

8.4 Additional Reporting Requirements for Serious Adverse Events

8.4.1 Investigator Reporting Responsibilities

Any Serious Adverse Event (SAE) must be reported to NEOX on the Serious Adverse Event Form – Clinical Trials within 24 hours of first knowledge. This report should contain an assessment of available information on seriousness, severity, causal relationship to the investigational product, comparator or trial procedure, the action taken, the outcome to date, and a narrative description of the course of the event.

The completed SAE form must be faxed or scanned and e-mailed to NEOX using the following fax number or e-mail address:

Fax number: +420 241 400 761

E-mail address: **pv-hamlet@neoxcro.com**

It may be relevant for the investigator to enclose other information with the SAE form, such as reports of diagnostic procedures, hospital records, autopsy reports, etc.

The investigator must notify the local IRB(s)/IEC(s) of SAEs as required by current applicable legislation for the Czech Republic.

SAEs occurring after the completion of the clinical trial including any protocol required post-treatment follow-up period should not be routinely sought or collected. However, such events should be reported to NEOX at **pv-hamlet@neoxcro.com** if the investigator becomes aware of them.

The criteria qualifying the AE to be an SAE should be recorded in the CRF (Appendix 3).

8.4.2 CRO Reporting Responsibilities

Global Pharmacovigilance at NEOX is responsible for assessing whether or not an SAE is expected. The relevant reference document is the Investigator's Brochure and subsequent updates.

Global Pharmacovigilance at NEOX will notify the regulatory authorities and concerned investigators of all relevant safety information according to the current applicable legislation for the concerned countries.

Notification of the IECs / IRBs about all relevant events (e.g. SAEs, SUSARs) will be performed by NEOX.

THIS DOCUMENT CONTAINS TRADE SECRETS, AND/OR COMMERCIAL INFORMATION, DELIVERED IN CONFIDENCE AND RELIANCE THAT SUCH INFORMATION WILL NOT BE COPIED OR MADE AVAILABLE TO ANY THIRD PARTY WITHOUT THE WRITTEN CONSENT OF HAMLET PHARMA AB.

In compliance with applicable regulations, in the event of a SUSAR, the subject's treatment code will usually be unblinded before reporting to the competent authorities, IECs/IRBs. For reporting to investigators the treatment will, if possible, be kept blind.

All SAEs that are assessed as causally related to the investigational product(s) by either the investigator or NEOX, and which are not expected (SUSARs) are subject to expedited reporting to regulatory authorities and IRB(s)/IEC(s) according to the current applicable legislation in the concerned countries. Investigators will be notified of these on an ongoing basis.

8.5 Follow-up for Final Outcome of Adverse Events

During the trial, the investigator should follow up for final outcome on all AEs (including SAEs). Once a subject leaves the clinical trial, the investigator should follow up on the outcome of all non-serious AEs classified as of possible/probable relationship to the investigational product for 14 ± 2 days or until the final outcome is determined, whichever comes first. SAEs must be followed up until a final outcome has been established, i.e. the follow-up may continue beyond the end of the clinical trial.

8.6 Assessments and Documentation of Adverse Events

Attention is to be paid to the time of occurrence of AEs at all stages of the examination. Thus, the subject should be closely observed by the investigator.

AEs observed, mentioned upon open questioning by a member of the investigator's team or spontaneously reported by the patient will be documented. The observation period for AEs will start with administration of first dose of study drug and will end with the last visit of follow-up **except** for AEs related to study procedures; the observation period for the latter AEs will start with signed informed consent and will end with the last visit of follow-up. In the event of ongoing study-related AEs and medically relevant AEs at the end of the study, the investigator is urged to monitor the subject and document the outcome on the subject's source document. After the end of the follow-up phase, there is no requirement to actively collect AEs including deaths.

The investigator is responsible for the grading of each category mentioned. An assessment of the **seriousness** of the event will be made by the investigator. All AEs will be recorded on the AE page of the CRF.

Emerging AEs will be allocated to the period in which they have started, e.g. a symptom starting in the treatment period and continuing in the follow-up period without deterioration will only be documented for that treatment period.

When assigning its cause, '*death*' should **not** be recorded as an AE on the AE page. Instead, '*death*' is the <u>outcome</u> of underlying AE(s).

THIS DOCUMENT CONTAINS TRADE SECRETS, AND/OR COMMERCIAL INFORMATION, DELIVERED IN CONFIDENCE AND RELIANCE THAT SUCH INFORMATION WILL NOT BE COPIED OR MADE AVAILABLE TO ANY THIRD PARTY WITHOUT THE WRITTEN CONSENT OF HAMLET PHARMA AB.

For all serious adverse events (SAEs), the sponsor has to carry out a separate assessment for expectedness, seriousness and causal relationship to study drug.

9 Investigational Product(s)

9.1 Description of Investigational Product

The active ingredient, α1H, consists of a chemically synthesized 39 amino acid peptide in complex with oleic acid in the ration 1:5. The peptide originates from the alpha1 domain of alpha-lactalbumin and the peptide sequence is KQFTKAELSQLLKDIDGYGGIALPELIATMFHTSGYDTQ and the molecular weight is 4344 Da.

Each vial of the IP, α1H solution for instillation, contains 34 mL and the concentration of the active ingredient is 7.4 mg/mL (1.7 mM) with respect to the peptide and 2.6 mg/mL (8.5 mM) with respect to the oleic acid. Each vial further contains sodium chloride, potassium chloride, sodium and potassium phosphate and water for injection. 30mL will be instilled per subject (14 F Catheter has a dead volume of 4 mL). One vial with corresponding placebo contains sodium chloride, potassium chloride, sodium and potassium phosphate and water for injection and is identical to the active treatment in appearance.

9.2 Manufacturing, packaging and labelling of the Investigational Product

α1H 7.4 mg/mL solution for instillation and corresponding placebo is manufactured, packaged and released for the trial in accordance with current Good Manufacturing Practice (cGMP).

The Investigational Product is supplied frozen in 50-mL glass vials. Vials for the treatment of one patient will be shipped in a secondary cardboard packaging.

The labelling of α1H 7.4 mg/mL solution for instillation and corresponding placebo will be according to EudraLex volume 4, Annex 13 *Good manufacturing practices for Medicinal products for human and veterinary use* and applicable local regulatory requirements. Active treatment and placebo will be blinded and distinguished by a blinded identifier (Vial code).

9.3 Storage and handling of the Investigational Product

The α1H 7.4 mg/mL solution for instillation and corresponding placebo will be stored in a freezer at a temperature of -20°C. The temperature of the freezer will be logged.

The IP will be stored at the trial site as required by local regulations and laws for the participating site.

THIS DOCUMENT CONTAINS TRADE SECRETS, AND/OR COMMERCIAL INFORMATION, DELIVERED IN CONFIDENCE AND RELIANCE THAT SUCH INFORMATION WILL NOT BE COPIED OR MADE AVAILABLE TO ANY THIRD PARTY WITHOUT THE WRITTEN CONSENT OF HAMLET PHARMA AB.

The IP, α1H 7.4 mg/mL solution for instillation will be thawed as described in the study specific lab manual. The handling of placebo is also described in the study specific lab manual.

9.4 Administration of Investigational Product

Route of administration	The total volume of thawed solution is used for instillation into the urine bladder.
Dosing frequency	One dose at 6 occasions during the trial.
Time of day for dosing	No specific requirements
Relation of time of dosing to dietary intake	No specific requirements
Relation of time of dosing to clinical assessments	Clinical assessments will be done 1-3 hours after each instillation. Final evaluation after 6 instillations.

9.5 Non-Investigational Medicinal Products

The marketed product, as available, will be used for this study without any re-labelling or re-packaging of the product. This will also include the pregnancy tests provided by the sponsor.

9.6 Drug Accountability and Compliance Checks

9.6.1 Drug Accountability Investigational Product

The investigator is fully responsible for the investigational products at the trial site, for maintaining adequate control of the investigational products, and for documenting all transactions with them.

An inventory (Individual Drug Accountability Form) must be kept tracking the investigational product given to each subject enrolled in the trial. This inventory must be available for inspection during monitoring visits and will be checked by the monitor to ensure correct dispensing of the investigational product.

9.6.2 Trial Product Destruction

All investigational products (used and unused) supplied by the Contract Manufacturing Organisation (CMO) on behalf of Hamlet Pharma will be destroyed by the local Pharmacy at the hospital. Before sending investigational products to the Pharmacy, they must be fully accounted for by the monitor with the help of the person responsible for dispensing the investigational products.

THIS DOCUMENT CONTAINS TRADE SECRETS, AND/OR COMMERCIAL INFORMATION, DELIVERED IN CONFIDENCE AND RELIANCE THAT SUCH INFORMATION WILL NOT BE COPIED OR MADE AVAILABLE TO ANY THIRD PARTY WITHOUT THE WRITTEN CONSENT OF HAMLET PHARMA AB.

9.7 Emergency Unblinding of Individual Subject Treatment

For each subject, an individual sealed envelope containing the randomisation code will be kept at the clinic (which is locked with restricted access) to enable the investigator to break the code, for safety purposes, if required during the conduct of the study. If it becomes necessary to break the code during the study, the date, time and reason will be recorded in the subject's source data and on the individual envelope. Every attempt should be made to contact the sponsor before un-blinding a subject.

10 Statistical Methods

10.1 Determination of Sample Size

The primary objective of the trial is to evaluate the safety of α1H. No formal sample size calculation evaluating the power of the trial has been performed. However, a consideration regarding the sample size was made as described below.

For efficacy, the sample size was based on analysis of change in tumor cells assessed before HAMLET instillation and 2 hours after it. The mean of fold increase of shed cells was 41.3 and standard deviation was 60.4 in 9 examined patients from the previous study. The number of required enrolled subjects is 20 cases in both groups to achieve criterion for significance (alpha) 0.05 and power 90% using the paired samples 1-tailed t-test. The null hypothesis is H0: mean change in cell shedding = 0 and the alternative hypothesis is HA: mean change in cell shedding > 0. Positive increase in tumor cells was confirmed in previous study.

10.2 Definition of Trial Analysis Sets

All subjects enrolled in the trial (i.e. subjects for whom informed consent has been obtained and who have been registered in a clinical trial) will be accounted for in the clinical trial report.

A full analysis set will comprise all enrolled subjects who meet the protocol-defined eligibility criteria.

A safety analysis set will be defined by excluding subjects from the full analysis set who either received no treatment with investigational product and/or for whom no post-baseline safety evaluations are available.

The decisions regarding inclusion/exclusion of subjects and/or subject data from the trial analysis sets will be documented in the clinical trial report.

THIS DOCUMENT CONTAINS TRADE SECRETS, AND/OR COMMERCIAL INFORMATION, DELIVERED IN CONFIDENCE AND RELIANCE THAT SUCH INFORMATION WILL NOT BE COPIED OR MADE AVAILABLE TO ANY THIRD PARTY WITHOUT THE WRITTEN CONSENT OF HAMLET PHARMA AB.

10.3 Statistical Analysis

10.3.1 Disposition of Subjects

The reasons for leaving the trial will be presented for all subjects assigned treatment by last visit attended.

10.3.2 Demographics and other Baseline Characteristics

Descriptive statistics of demographics and other baseline characteristics will be presented for all subjects assigned treatment and separately for per protocol analysis set.

Demographics include age, sex, race and ethnicity. Other baseline characteristics include height, weight and BMI, concurrent diagnoses (from medical history and indications for concomitant medication) and concomitant medication.

10.3.3 Analysis of Primary Endpoints

Adverse events

The number and percentage of subjects experiencing each type of adverse event will be tabulated for the safety analysis set. Further details are included in section 10.3.5.1.

Quantification of cell shedding in urine

The total number of epithelial cells in a unit (in mL) of urine, will be analysed. The change in cell shedding comparison will be made:

- intra-individually (paired test) of each visit and longitudinal data during study period.
- group-wise (two-samples) between active drug and placebo group at Visit 1-6.

The ratio of live and dead cells will be analysed in a unit (in mL) of urine, totally and for the malignant or non-malignant cells at Visit 1-6 (paired test).

Characteristics of papillary tumors

Continuous variables, the number of papillary tumors and a change in papillary tumor shape and size will be analysed at Visit 1 and 7 (paired test).

10.3.4 Analysis of Secondary Endpoints

The analysis of secondary endpoints will be based on the full analysis set.

THIS DOCUMENT CONTAINS TRADE SECRETS, AND/OR COMMERCIAL INFORMATION, DELIVERED IN CONFIDENCE AND RELIANCE THAT SUCH INFORMATION WILL NOT BE COPIED OR MADE AVAILABLE TO ANY THIRD PARTY WITHOUT THE WRITTEN CONSENT OF HAMLET PHARMA AB.

Histopathology scoring

Categorical variables, the grade and the stage/invasiveness of tissue changes will be summarised at Visit 1 and 7.

Continuous variables, the tumor markers, cell death, proliferation, and inflammation of change in tumor tissue will be summarised at Visit 1 and 7.

Tissue accumulation of α1H

The tumor-specific uptake of α1H by tumor tissue will be compared to healthy tissue at Visit 7.

Tumor response to α1H by gene expression analysis

Gene expression analysis will be compared group-wise (two-samples) between active drug and placebo group at Visit 7.

Urine cytology and apoptosis

The grade and the stage/invasiveness of cell changes in urine, apoptotic changes in shed cells, and other markers of cell death will be summarised at Visit 1 and 7.

Proteomic analysis of markers in urine

The change in protein markers will be analysed intra-individually at each visit and group-wise at Visit 1, 7 and F-U 1.

Long-term effect of the study treatment

Progression or non-progression of the study disease during the extended follow-up period of 24 months.

Time to first recurrence i.e. time between TURB (Visit 7) to next TURB, within the extended follow-up period of 24 months.

10.3.5 Analysis of Safety

The analysis of safety will be based on the safety analysis set, except where otherwise stated.

THIS DOCUMENT CONTAINS TRADE SECRETS, AND/OR COMMERCIAL INFORMATION, DELIVERED IN CONFIDENCE AND RELIANCE THAT SUCH INFORMATION WILL NOT BE COPIED OR MADE AVAILABLE TO ANY THIRD PARTY WITHOUT THE WRITTEN CONSENT OF HAMLET PHARMA AB.

10.3.5.1 Adverse Events

Adverse events will be coded during the course of the trial according to MedDRA. Adverse events reported up to 30 days after last active treatment/placebo administration will be presented by preferred terms and primary system organ class.

Treatment emergent AEs will be summarised, however all adverse events recorded during the course of the trial will be included in the subject data listings. An event will be considered emergent with the trial treatment if started after the first application of investigational product or if started before the first application of investigational product (applicable if subject had a wash-out) and worsened in severity thereafter. The tabulations described in the following will only include the events that are emergent with trial treatment. In each of the tabulations, adverse events are defined by MedDRA preferred terms within primary system organ class.

An overall summary of the number (percentage) of subjects with any treatment emergent AEs, SAEs, premature discontinuations from the trial due to AEs, treatment related AEs and severe AEs will be presented.

The number of subjects experiencing each type of adverse events will be tabulated regardless of the number of times each adverse event is reported by each subject.

The severity for each type of adverse event will be tabulated. Where there are several recordings of severity for a given type of adverse event, severity will be taken as the most severe recording for that adverse event.

The causal relationship to trial medication for each type of adverse events will be tabulated. Where there are several recordings of causal relationship to the investigational product for a given type of adverse event, causal relationship will be taken as the most-related recording from the last report of that adverse event, since that is when the (sub)investigator will be in possession of most information and so best able to judge causal relationship.

Related adverse events are defined as adverse events for which the (sub)investigator has not described the causal relationship to investigational product as 'not related'. The number of subjects experiencing each type of related adverse event will be tabulated regardless of the number of times each related adverse event is reported by each subject.

The number of subjects experiencing each type of lesional/perilesional adverse event, lesional/perilesional adverse event on the body and lesional/perilesional adverse event on the scalp will be tabulated.

Serious adverse events will be evaluated separately and a narrative for each will be given.

THIS DOCUMENT CONTAINS TRADE SECRETS, AND/OR COMMERCIAL INFORMATION, DELIVERED IN CONFIDENCE AND RELIANCE THAT SUCH INFORMATION WILL NOT BE COPIED OR MADE AVAILABLE TO ANY THIRD PARTY WITHOUT THE WRITTEN CONSENT OF HAMLET PHARMA AB.

AEs leading to withdrawal from trial or discontinuation of investigational product will be listed.

10.3.5.2 Vital Signs

For vital signs (systolic and diastolic blood pressure and heart rate), the absolute value by visit and change from baseline (Visit 1) to Visit 7 will be summarised.

Clinically significant abnormalities in the vital signs at Visit 1 and Visit 7 will be presented. Any abnormalities at Visit 7 were not present at baseline will be highlighted.

10.3.5.3 Clinical Laboratory Evaluation

For biochemistry and haematology parameters, the absolute value and the change from baseline to Visit 7 and baseline to F-U 1 Visit will be summarised. In addition, the laboratory parameters will be classified as 'low', 'normal' or 'high', depending on whether the value is below, within or above the reference range, respectively. Shift tables will be produced showing the categories at baseline against those at Visit 7 and baseline to F-U 1 Visit.

Urinalysis parameters measured in spot urine will be analysed and presented as outlined above for the biochemistry and haematology parameters.

For the urinary glucose and ketones, the values will be categorised as absent or present. Shift tables will be produced showing the presence/absence at baseline against presence/absence at Visit 7 and baseline to F-U 1 Visit.

10.3.6Interim Analysis

Interim analysis is planned after completion of the main study, i.e. after all patients have completed visit FU-1 30 days after treatment termination. Interim analysis will be performed to the extent described under

10.3.7 General Principles

All confidence intervals will be presented with 95% degree of confidence.

An observed cases approach will be used for tabulations of data by visit (i.e. involving only those subjects who attended each specific visit).

The end of treatment values will be presented for laboratory parameters (primary and secondary endpoints) and for efficacy data.

For tabulations on changes from baseline, baseline will be defined as the last assessment performed before application of α1H.

THIS DOCUMENT CONTAINS TRADE SECRETS, AND/OR COMMERCIAL INFORMATION, DELIVERED IN CONFIDENCE AND RELIANCE THAT SUCH INFORMATION WILL NOT BE COPIED OR MADE AVAILABLE TO ANY THIRD PARTY WITHOUT THE WRITTEN CONSENT OF HAMLET PHARMA AB.

Categorical data will be summarised using the number and percentage of subjects in each category. Continuous data will be summarised using the mean, 95% confidence interval for mean (CI), standard deviation (SD), median, minimum and maximum values.

Any changes from the statistical analysis planned in this clinical trial protocol will be described and justified in a protocol amendment and/or in the clinical trial report dependent on the type of deviation.

The main study will end and will be evaluated during Interim analysis to the extent described in section 10.3. Final analysis will occur after all patients participating in the Extended Follow-up Part have completed the 24 months follow-up. During this analysis long term effect of the study treatment will be analyzed as described under 10.3.4

11 Case Report Forms and Data Handling

11.1 Case Report Forms (CRFs)

Data will be collected by means of Electronic Data Capture (EDC) for the main study. Data collected during the extended Follow-up period will not be captured in the EDC system. These data will be reported in a separate paper template. The investigator or staff authorised by the investigator must enter subject data into electronic CRFs. Data recorded in the electronic CRFs must be accessible to site staff through a secure internet connection immediately after entry. The CRFs must be maintained in an up-to-date condition at all times.

The investigator must electronically sign all CRFs used. This signature information (including date of signature) will be kept in the audit trail and cannot be altered. Any correction(s) made by the investigator or authorised site staff to the CRF after original entry will be documented in the audit trail. Previously approved changes to the data, will require the re-signature of the investigator. The person making the change and the date, time and reason for the change will be identified in the audit trail.

For archiving purposes, each investigator must be supplied with a copy of the CRFs for all subjects enrolled at the trial site via an electronic medium at completion of the trial and before access to the eCRF is revoked. Audit trail information must be included. CRFs must be available for inspection by authorised representatives from NEOX (e.g. audit by the quality assurance department), from regulatory authorities and/or IEC/IRBs.

THIS DOCUMENT CONTAINS TRADE SECRETS, AND/OR COMMERCIAL INFORMATION, DELIVERED IN CONFIDENCE AND RELIANCE THAT SUCH INFORMATION WILL NOT BE COPIED OR MADE AVAILABLE TO ANY THIRD PARTY WITHOUT THE WRITTEN CONSENT OF HAMLET PHARMA AB.

11.2 Data Handling

Subject data should be entered into the eCRF as soon as possible after the visit in accordance with the time requirements described in the Clinical Trial Agreement with the site. Queries for discrepant data may be generated automatically by the system upon entry or generated manually by the monitor or the trial data manager. All queries, whether generated by the system or by a user, will be in an electronic format. This systematic validation will ensure that a clean and consistent database is provided prior to the statistical analysis being performed.

11.3 Source Data

For all data recorded, the source document must be defined in a source document agreement at the trial site. There must only be one source defined at any time for any data elements.

The trial monitor will check the CRFs for accuracy and completeness by verifying data recorded in the CRF against source data to ensure such records are consistent.

Source data should, as a general rule be recorded in the subject's medical record or other defined document normally used at the trial site. Source data not normally collected as a routine part of the clinical practice at the site may be entered on a worksheet. Clinical assessments/safety evaluations must be signed by medically qualified (sub)investigators.

If the worksheet does not become part of the subject's medical record, the following should as a minimum be added to the subject's medical record:

For this clinical trial, the following parameters collected in the CRF should be verifiable from source documents available at the trial site:

- Date of trial visits and date leaving the clinical trial
- Relevant medical history and diagnosis
- Nature of contraception used by the subject and result of pregnancy test(s), when applicable
- Data for evaluation of eligibility criteria
- Dispensation/administration of investigational product
- Non-investigational medicinal products used for each subject
- Concomitant medication (including changes) and diagnoses
- Subject demographics (sex, date of birth, race, ethnic origin)
- Clinical assessments (vital signs, physical examination, investigator's assessments)
- Laboratory assessments (haematology, biochemistry and urine tests)
- Adverse events, (nature, dates)

THIS DOCUMENT CONTAINS TRADE SECRETS, AND/OR COMMERCIAL INFORMATION, DELIVERED IN CONFIDENCE AND RELIANCE THAT SUCH INFORMATION WILL NOT BE COPIED OR MADE AVAILABLE TO ANY THIRD PARTY WITHOUT THE WRITTEN CONSENT OF HAMLET PHARMA AB.

In addition to the above, the following should be added to the subject's medical record in chronological order:

- Date(s) of conducting the informed consent process including date of provision of subject information
- Subject screening number
- Subject randomisation number
- Investigational product Vial code
- The fact that the subject is participating in a clinical trial in bladder cancer
- Other relevant medical information

11.4 Trial Monitoring

During the course of the trial, the monitor will visit the trial site to ensure that the protocol and GCP are adhered to, that all issues have been recorded to perform source data verification and to monitor drug accountability

The first monitoring visit should be performed as soon as possible after Visit 1 and no later than 2 weeks after.

The monitoring visit intervals will depend on the trial site's recruitment rate, the compliance of the trial site with the protocol and GCP.

In order to perform their role effectively, monitors and persons involved in quality assurance and inspections will need direct access to source data, e.g. medical records, laboratory reports, appointment books, etc. If the electronic medical record does not have a visible audit trail, the investigator must provide the monitor with signed and dated printouts. In addition, relevant site staff should be available for discussions at monitoring visits and between monitoring visits (e.g. by telephone).

The extended follow up period will be monitored to less extent. Monitoring will mainly be done through telephone contacts. If any quality issue should arise the monitor will pay a visit to the site. All PIC for the extension part will be sourced verified.

12 Handling of an Urgent Safety Measure

An Urgent Safety Measure is a measure taken to implement an action/protocol deviation under an emergency. This is defined within the EU Directive as *"…the occurrence of any new event relating to the conduct of the trial or the development of the investigational medicinal product*

THIS DOCUMENT CONTAINS TRADE SECRETS, AND/OR COMMERCIAL INFORMATION, DELIVERED IN CONFIDENCE AND RELIANCE THAT SUCH INFORMATION WILL NOT BE COPIED OR MADE AVAILABLE TO ANY THIRD PARTY WITHOUT THE WRITTEN CONSENT OF HAMLET PHARMA AB.

where that new event is likely to affect the safety of the subjects, the sponsor and the investigator shall take appropriate urgent safety measures to protect the subjects against any immediate hazard." (Article 10(b) of Directive 2001/20/EC).

If the investigator becomes aware of information that necessitates an immediate change in the clinical trial procedure or a temporary halt to the clinical trial in order to protect clinical trial subjects from any immediate hazard to their health and safety, the investigator can do so without prior approval from Hamlet Pharma, regulatory authority(ies) or IRB(s)/IEC(s).

The investigator must immediately inform Hamlet Pharma - by contacting Professor Catharina Svanborg - of this change in the clinical trial procedure or of the temporary halt providing full details of the information and the decision-making process leading to the implementation of the urgent safety measure.

13 Quality Assurance/Audit

The clinical trial will be subject to audits conducted by NEOX or inspections from domestic or foreign regulatory authorities or from IRBs/IECs. Audits and inspections may take place during or after the trial. The investigator and the site staff as well as NEOX staff have an obligation to cooperate and assist in audits and inspections. This includes giving auditors and inspectors direct access to all source documents and other documents at the trial site relevant to the clinical trial. This includes permission to examine, verify and reproduce any records and reports that are important to the evaluation of the trial.

If the trial site is contacted for an inspection by competent authorities, both NEOX and Hamlet Pharma must be notified immediately.

14 Completion of Trial

14.1 Criteria for Premature Termination of the Trial and/or Trial Site

Hamlet Pharma, the investigator, the IRB/IECs or competent authorities may decide to stop the trial, part of the trial or a trial site at any time, but agreement on procedures to be followed must be obtained.

If a trial is suspended or prematurely terminated, the investigator must inform the subjects promptly and ensure appropriate therapy and follow-up. As specified by applicable regulatory requirements, either the investigator or Hamlet Pharma must promptly inform IRB/IECs and provide a detailed written explanation. Relevant competent authorities must be informed.

THIS DOCUMENT CONTAINS TRADE SECRETS, AND/OR COMMERCIAL INFORMATION, DELIVERED IN CONFIDENCE AND RELIANCE THAT SUCH INFORMATION WILL NOT BE COPIED OR MADE AVAILABLE TO ANY THIRD PARTY WITHOUT THE WRITTEN CONSENT OF HAMLET PHARMA AB.

The trial must be terminated if the perception of the benefit/risk ratio (judged from clinical signs and symptoms, (S)AEs and/or remarkable safety laboratory changes) becomes unfavourable for the continuation of the trial.

14.2 Provision for Subject Care Following Trial Completion

After the completion of the trial, the subjects will be treated at the investigator's discretion or referred to other physician(s) according to standard practice.

14.3 Archiving of Trial Documents

The investigator at each trial site must make arrangements to store essential trial documents, including the Investigator Trial File (ICH E6, Guideline for Good Clinical Practice) until Hamlet Pharma informs the investigator that the documents are no longer to be retained or longer if required by local regulations.

In addition, the investigator is responsible for the archiving of all relevant source documents so that the trial data can be compared against source data after the completion of the trial (e.g. in case of an inspection from regulatory authorities).

The investigator is required to ensure the continued storage of the documents even if the investigator leaves the clinic/practice or retires before the end of the required storage period.

The destruction process must ensure confidentiality of data and must be done in accordance with local regulatory requirements.

15 Ethics and Regulatory Authorities

15.1 Institutional Review Boards (IRBs)/Independent Ethics Committees (IECs) and Regulatory Authorities

Written approval or favourable opinion must be obtained from relevant IRB/IECs prior to the enrolment of subjects.

Any amendments to the approved clinical trial must be approved by/receive favourable opinion from relevant IRBs/IECs and regulatory authorities as required prior to the implementation.

The appropriate regulatory authority must approve the clinical trial, as required.

THIS DOCUMENT CONTAINS TRADE SECRETS, AND/OR COMMERCIAL INFORMATION, DELIVERED IN CONFIDENCE AND RELIANCE THAT SUCH INFORMATION WILL NOT BE COPIED OR MADE AVAILABLE TO ANY THIRD PARTY WITHOUT THE WRITTEN CONSENT OF HAMLET PHARMA AB.

15.2 Ethical Conduct of the Trial

This clinical trial must be conducted in accordance with the principles of the current revision at the start of the trial of the World Medical Association (WMA), Declaration of Helsinki, Ethical Principles for Medical Research Involving Human Subjects.

15.3 Patient Information and Informed Consent

The subject's signed and dated informed consent to participate in the clinical trial must be obtained prior to any clinical trial related procedure being carried out in accordance with ICH GCP (4.8) and all applicable laws and regulations.

The parent(s)/legal guardian(s) of subjects will receive written and verbal information concerning the trial or subjects may give informed consent as appropriate and according to national laws or regulations. This information will emphasise that participation in the trial is voluntary and that the subject may withdraw from the trial at any time and for any reason. The parent(s)/legal guardian(s) will be given an opportunity to ask questions and will be given sufficient time to consider before consenting. Signed and dated informed consent for the subject to participate in the trial will be obtained from the parent(s)/legal guardian(s), or by the subject in accordance with national laws or regulations, prior to any trial related procedure being carried out.

All subjects will also receive appropriate written and verbal information, be given an opportunity to ask questions and sufficient time to consider, before providing written assent. The subject's decision not to participate or to withdraw will be respected, even if consent is given by the parent(s)/legal guardian(s).

The subject's signed and dated informed consent to participate in the clinical trial must be obtained prior to any clinical trial related procedure being carried out in accordance with ICH GCP (4.8) and all applicable laws and regulations.

Participation in the optional extended follow-up period will be offered to all subjects participating in the main study. To take part in the optional extended follow up period patients will need to sign a separate informed consent form, that allow the study site to collect information from the patients' routine care visits and report the data into the study as described in section 7.17.

THIS DOCUMENT CONTAINS TRADE SECRETS, AND/OR COMMERCIAL INFORMATION, DELIVERED IN CONFIDENCE AND RELIANCE THAT SUCH INFORMATION WILL NOT BE COPIED OR MADE AVAILABLE TO ANY THIRD PARTY WITHOUT THE WRITTEN CONSENT OF HAMLET PHARMA AB.

15.4 Processing of Personal Data

This protocol specifies the personal data on trial subjects (e.g. age, gender, health condition, height, medical history, test results, etc.) which shall be collected as part of the trial and processed during and after trial completion.

Investigators and Hamlet Pharma must ensure that collection, processing and transfer of personal data are in compliance with national legislation on data protection and privacy.

16 Insurance

Hamlet Pharma has taken out relevant insurances covering the subjects in the present clinical trial in accordance with applicable laws and regulations.

17 Use of Information

This clinical trial protocol as well as all other information, data and results relating to this clinical trial and/or to the investigational product(s) is confidential information belonging to Hamlet Pharma and shall not be used by the investigator for purposes other than this clinical trial.

The investigator agrees that Hamlet Pharma may use any and all information, data and results from this clinical trial in connection with the development of the investigational product(s) and, therefore, may disclose and/or transfer information, data and/or results to other investigators, regulatory authorities and/or commercial partners.

18 Publication

Basic information of this clinical trial will be posted on the website: www.clinicaltrials.gov before the first subject enters into the clinical trial.

Any publication of data will be prepared in collaboration between Hamlet Pharma and the International Coordinating Investigator and approved by both parties before publication.

19 Responsibilities

The International Coordinating Investigator (ICI) is responsible for the approval of the (Consolidated) Clinical Trial Protocol, Clinical Trial Protocol Amendment(s) and the Clinical Trial Report on behalf of all clinical trial investigators.

Each participating investigator is responsible for all aspects of the clinical trial conduct.

THIS DOCUMENT CONTAINS TRADE SECRETS, AND/OR COMMERCIAL INFORMATION, DELIVERED IN CONFIDENCE AND RELIANCE THAT SUCH INFORMATION WILL NOT BE COPIED OR MADE AVAILABLE TO ANY THIRD PARTY WITHOUT THE WRITTEN CONSENT OF HAMLET PHARMA AB.

20 List of Abbreviations

AE	Adverse Event
ALP	Alkaline Phosphatase
AUC	Area Under Curve
CDMS	Clinical Data Management System
cGMP	Current Good Manufacturing Practice
CI	Confidence Interval for mean
CMO	Contract Manufacturing Organisation
CRF	Case Report Form
CRO	Contract Research Organisation
CTCAE	Common Terminology Criteria for Adverse Events
EDC	Electronic Data Capture
EU	European Union
FDA	Food and Drug Administration
FSI	First Subject In
GCP	Good Clinical Practice
IB	Investigator´s Brochure
ICH	International Conference on Harmonisation
ID	Identification
IMP	Investigational Medicinal Product
IEC	Independent Ethics Committee
IRB	Institutional Review Board
LSO	Last Subject Out
NMIBC	Non-Muscle Invasive Bladder Cancer
SAE	Serious Adverse Event
SOP	Standard Operating Procedure
SPM	Study Procedures Manual
SUSAR	Suspected Unexpected Serious Adverse Reaction
VMA	World Medical Association
TURB	Trans Urethral Resection and Biopsy

THIS DOCUMENT CONTAINS TRADE SECRETS, AND/OR COMMERCIAL INFORMATION, DELIVERED IN CONFIDENCE AND RELIANCE THAT SUCH INFORMATION WILL NOT BE COPIED OR MADE AVAILABLE TO ANY THIRD PARTY WITHOUT THE WRITTEN CONSENT OF HAMLET PHARMA AB.

21 References

1. Tumörregistret, R., *Nationellt kvalitetsregister för blåscancer*. Universitetsjukhuset i Lund: 221 85 Lund.
2. Soloway, M.S., M. Sofer, and A. Vaidya, *Contemporary management of stage T1 transitional cell carcinoma of the bladder.* J Urol, 2002. **167**(4): p. 1573-83.
3. Sylvester, R.J., et al., *Predicting recurrence and progression in individual patients with stage Ta T1 bladder cancer using EORTC risk tables: a combined analysis of 2596 patients from seven EORTC trials.* Eur Urol, 2006. **49**(3): p. 466-5; discussion 475-7.
4. Aldousari, S. and W. Kassouf, *Update on the management of non-muscle invasive bladder cancer.* Can Urol Assoc J, 2010. **4**(1): p. 56-64.
5. Nieuwenhuijzen, J.A., A. Bex, and S. Horenblas, *Unusual complication after immediate postoperative intravesical mitomycin C instillation.* Eur Urol, 2003. **43**(6): p. 711-2.
6. Malmstrom, P.U., *Advances in intravesical therapy of urinary bladder cancer.* Expert Rev Anticancer Ther, 2004. **4**(6): p. 1057-67.
7. Schenkman, E. and D.L. Lamm, *Superficial bladder cancer therapy.* ScientificWorldJournal, 2004. **4 Suppl 1**: p. 387-99.
8. Argiris, K., C. Panethymitaki, and M. Tavassoli, *Naturally occurring, tumor-specific, therapeutic proteins.* Exp Biol Med (Maywood), 2011. **236**(5): p. 524-36.
9. Sethi, N. and Y. Kang, *Unravelling the complexity of metastasis - molecular understanding and targeted therapies.* Nat Rev Cancer, 2011. **11**(10): p. 735-48.
10. Hakansson, A., et al., *Apoptosis induced by a human milk protein.* Proc Natl Acad Sci U S A, 1995. **92**(17): p. 8064-8.
11. Svensson, M., et al., *Conversion of alpha-lactalbumin to a protein inducing apoptosis.* Proc Natl Acad Sci U S A, 2000. **97**(8): p. 4221-6.
12. Fischer W , L., et al., - *Human alpha-lactalbumin made lethal to tumor cells (HAMLET) kills human glioblastoma cells in brain xenografts by an apoptosis-like mechanism and prolongs survival.* Cancer Res, 2004. **64**(6): p. 2105-12.
13. Mossberg, A.K., et al., *HAMLET interacts with lipid membranes and perturbs their structure and integrity.* PLoS One, 2010. **5**(2): p. e9384.
14. Storm, P., et al., *Conserved features of cancer cells define their sensitivity to HAMLET-induced death; c-Myc and glycolysis.* Oncogene, 2011. **30**(48): p. 4765-79.
15. Rammer, P., et al., *BAMLET activates a lysosomal cell death program in cancer cells.* Mol Cancer Ther, 2010. **9**(1): p. 24-32.
16. Permyakov, S.E., et al., *Oleic acid is a key cytotoxic component of HAMLET-like complexes.* Biol Chem, 2012. **393**(1-2): p. 85-92.
17. Permyakov, S.E., et al., *A novel method for preparation of HAMLET-like protein complexes.* Biochimie, 2011. **93**(9): p. 1495-501.
18. Xie, Y., et al., *Electrostatic interactions play an essential role in the binding of oleic acid with alpha-lactalbumin in the HAMLET-like complex: a study using charge-specific chemical modifications.* Proteins, 2013. **81**(1): p. 1-17.
19. Gustafsson, L., et al., *Treatment of skin papillomas with topical alpha-lactalbumin-oleic acid.* N Engl J Med, 2004. **350**(26): p. 2663-72.
20. Mossberg, A.K., et al., *Bladder cancers respond to intravesical instillation of HAMLET (human alpha-lactalbumin made lethal to tumor cells).* Int J Cancer, 2007. **121**(6): p. 1352-9.
21. Puthia, M., et al., *Prevention and treatment of colon cancer by peroral administration of HAMLET (human alpha-lactalbumin made lethal to tumour cells).* Gut, 2013.
22. Nadeem, A., et al., *A new class of cancer therapeutics - Alpha-helical peptides form tumoricidal lipid complexes. Submitted..*
23. Storm P Fau - Aits, S., et al., *Conserved features of cancer cells define their sensitivity to HAMLET-induced death; c-Myc and glycolysis.* Oncogene, 2011. **30**(48): p. 4765-79.
24. Mossberg, A.K., et al., *HAMLET treatment delays bladder cancer development.* J Urol, 2010. **183**(4): p. 1590-7.
25. Puthia, M., et al., *Prevention and treatment of colon cancer by peroral administration of HAMLET (human alpha-lactalbumin made lethal to tumour cells).* Gut, 2013. **63**(1): p. 131-42.

THIS DOCUMENT CONTAINS TRADE SECRETS, AND/OR COMMERCIAL INFORMATION, DELIVERED IN CONFIDENCE AND RELIANCE THAT SUCH INFORMATION WILL NOT BE COPIED OR MADE AVAILABLE TO ANY THIRD PARTY WITHOUT THE WRITTEN CONSENT OF HAMLET PHARMA AB.

26. Fischer, W., et al., *Human alpha-lactalbumin made lethal to tumor cells (HAMLET) kills human glioblastoma cells in brain xenografts by an apoptosis-like mechanism and prolongs survival.* Cancer Res, 2004. **64**(6): p. 2105-12.
27. zur Hausen, H., *Papillomavirus infections--a major cause of human cancers.* Biochim Biophys Acta, 1996. **1288**(2): p. F55-78.
28. Huncharek, M. and B. Kupelnick, *Impact of intravesical chemotherapy versus BCG immunotherapy on recurrence of superficial transitional cell carcinoma of the bladder: metaanalytic reevaluation.* Am J Clin Oncol, 2003. **26**(4): p. 402-7.
29. de Villiers, E.M., *Papillomavirus and HPV typing.* Clin Dermatol, 1997. **15**(2): p. 199-206.
30. Gibbs, S., et al., *Local treatments for cutaneous warts: systematic review.* Bmj, 2002. **325**(7362): p. 461.
31. Leman, J.A. and E.C. Benton, *Verrucas. Guidelines for management.* Am J Clin Dermatol, 2000. **1**(3): p. 143-9.
32. Harwood, C.A., et al., *Human papillomavirus infection and non-melanoma skin cancer in immunosuppressed and immunocompetent individuals.* J Med Virol, 2000. **61**(3): p. 289-97.

THIS DOCUMENT CONTAINS TRADE SECRETS, AND/OR COMMERCIAL INFORMATION, DELIVERED IN CONFIDENCE AND RELIANCE THAT SUCH INFORMATION WILL NOT BE COPIED OR MADE AVAILABLE TO ANY THIRD PARTY WITHOUT THE WRITTEN CONSENT OF HAMLET PHARMA AB.
