# Supplementary material for: Bladder cancer therapy using a conformationally fluid tumoricidal peptide complex
Source: Nat Commun. 2021 Jun 8;12:3427. doi: 10.1038/s41467-021-23748-y (PMC8187399; doi:10.1038/s41467-021-23748-y)
Supplement: Supplementary file 3 — Reporting summary [file 41467_2021_23748_MOESM3_ESM.pdf]

nature research

Corresponding author(s): C. Svanborg

Last updated by author(s): Apr 15, 2021

Reporting Summary

Nature Research wishes to improve the reproducibility of the work that we publish. This form provides structure for consistency and transparency in reporting. For further information on Nature Research policies, see our Editorial Policies and the Editorial Policy Checklist.

Statistics

For all statistical analyses, confirm that the following items are present in the figure legend, table legend, main text, or Methods section.

n/a	Confirmed	
☐	☒	The exact sample size (*n*) for each experimental group/condition, given as a discrete number and unit of measurement
☐	☒	A statement on whether measurements were taken from distinct samples or whether the same sample was measured repeatedly
☐	☒	The statistical test(s) used AND whether they are one- or two-sided<br>*Only common tests should be described solely by name; describe more complex techniques in the Methods section.*
☒	☐	A description of all covariates tested
☐	☒	A description of any assumptions or corrections, such as tests of normality and adjustment for multiple comparisons
☐	☒	A full description of the statistical parameters including central tendency (e.g. means) or other basic estimates (e.g. regression coefficient) AND variation (e.g. standard deviation) or associated estimates of uncertainty (e.g. confidence intervals)
☐	☒	For null hypothesis testing, the test statistic (e.g. *F*, *t*, *r*) with confidence intervals, effect sizes, degrees of freedom and *P* value noted<br>*Give P values as exact values whenever suitable.*
☒	☐	For Bayesian analysis, information on the choice of priors and Markov chain Monte Carlo settings
☐	☒	For hierarchical and complex designs, identification of the appropriate level for tests and full reporting of outcomes
☐	☒	Estimates of effect sizes (e.g. Cohen's *d*, Pearson's *r*), indicating how they were calculated

Our web collection on statistics for biologists contains articles on many of the points above.

Software and code

Policy information about availability of computer code

Data collection

Model building: Discovery Studio 4.1 (Accelrys)
Molecular dynamics simulations: molecular dynamics package33 with Plumed 2.3 plugin (Gromacs 5.1.2)
Confocal microscopy - Laser scanning microscope (LSM) 510 or LSM 800 (both from Zeiss).
Light microscopy - Zen lite 2012 (Blue edition) Version 1.1.2.0 (Zeiss).
Dionex Ultimate HPLC 3000 Standard System
RNAseq system - NextSeq 500/550 System (Illumina)
Tecan Infinite F200 Microplate reader - i-control Version 1.6.19.2 (Tecan)
VNMRJ version 4.0 (Agilent Technologies)

Data analysis

Statistics: Prism version 6.02 (GraphPad Software)
Analysis of simulation data: RESP method of AmberTools16 (AMBER 2016); Gromacs tools (Gromacs package33); gmx cluster tool(Gromacs 5.1.2)
Analysis of images: ImageJ versions 1.47v to 1.52e (National Institutes of Health, USA)
Transcriptomic data: Partek express data (Partek); Transcriptome Analysis console (TAC) version 4.0.1.36. (Applied Biosystems); Ingenuity Pathway Analysis Version 57662101 (Qiagen)
Analysis of HPLC data: Chromeleon 6 software
Gitools version 2.1 (Biomedical Genomics Group, Barcelona, Spain)
R (Version 3.4) and the packages limma and DESeq2
Microsoft Excel for Mac Version 16.43
The detailed information for data analysis is described in the Method section.

For manuscripts utilizing custom algorithms or software that are central to the research but not yet described in published literature, software must be made available to editors and reviewers. We strongly encourage code deposition in a community repository (e.g. GitHub). See the Nature Research guidelines for submitting code & software for further information.

Data

Policy information about availability of data

All manuscripts must include a data availability statement. This statement should provide the following information, where applicable:

- Accession codes, unique identifiers, or web links for publicly available datasets
- A list of figures that have associated raw data
- A description of any restrictions on data availability

The data supporting the structural and cellular findings of this study are available within the article and its supplementary information files. The structural data referenced during the study are available in a public repository from the Protein Data Bank website (www.rcsb.org, DOI:10.2210/pdb1B9O/pdb, DOI:10.2210/pdb1R7G/pdb). The RNA sequencing data generated in this study have been deposited in the Gene Expression Omnibus (GEO) database under accession number GSE172112 [https://www.ncbi.nlm.nih.gov/geo/query/acc.cgi?acc=GSE172112]. Source data underlying Figures 1, 2, 5 and 6, and Supplementary Figures 1-9 are provided as a Source Data file.

Field-specific reporting

Please select the one below that is the best fit for your research. If you are not sure, read the appropriate sections before making your selection.

[x] Life sciences [ ] Behavioural & social sciences [ ] Ecological, evolutionary & environmental sciences

For a reference copy of the document with all sections, see nature.com/documents/nr-reporting-summary-flat.pdf

Life sciences study design

All studies must disclose on these points even when the disclosure is negative.

Sample size	Determination of Sample Size<br>The primary objective of the trial was to evaluate the safety of alpha1-oleate. No formal sample size calculation evaluating the power of the trial has been performed. However, a consideration regarding the sample size was made based on a previous open study of HAMLET instillations in bladder cancer patients and in the murine bladder cancer model. For efficacy, the sample size was based on analysis of change in tumor cells assessed before HAMLET instillation and after 2 hours. The mean fold increase of shed cells was 41.3 and standard deviation was 60.4 in 9 patients16. A sample size of 20 patients per group was deemed suitable to achieve criterion for significance (alpha) 0.05 and power 90% using the paired samples 1-tailed t-test. The null hypothesis is H0: mean change in cell shedding = 0 and the alternative hypothesis is HA: mean change in cell shedding > 0.<br><br>For in vitro data no sample size calculations were performed before experiments but at least two repeated experiments were performed by independent researchers.
Data exclusions	No patient was excluded. Two data points were excluded from the analysis of TUNEL staining due to the medical conditions from patients and this was also confirmed by the Grubbs's outlier statistical test.
Replication	Replication of clinical study was not relevant. All in vitro experiments were performed at least twice by independent researchers.
Randomization	The clinical study was randomized by a third party through the study monitoring organization and placebo controlled.<br>For experiments other than those involving human participants, no animal studies were involved.<br>Randomization was not relevant to the cell culture study. The commercially available cell lines are homogeneous.
Blinding	The clinical study was double blind and unblinded only after each patient had completed the 52 day safety follow up and after data lock.<br>For experiments other than those involving human participants, the experiments were not blinded but at least two repeated experiments were performed by independent researchers.

Reporting for specific materials, systems and methods

We require information from authors about some types of materials, experimental systems and methods used in many studies. Here, indicate whether each material, system or method listed is relevant to your study. If you are not sure if a list item applies to your research, read the appropriate section before selecting a response.

Materials & experimental systems

n/a	Involved in the study
☐	☒ Antibodies
☐	☒ Eukaryotic cell lines
☒	☐ Palaeontology and archaeology
☒	☐ Animals and other organisms
☐	☒ Human research participants
☐	☒ Clinical data
☒	☐ Dual use research of concern

Methods

n/a	Involved in the study
☒	☐ ChIP-seq
☒	☐ Flow cytometry
☒	☐ MRI-based neuroimaging

Antibodies

Antibodies used

Rabbit polyclonal anti-human alpha lactalbumin (Mybiosource, Cat# MBS175270)
Goat anti-rabbit-Alexa Fluor 568 (ThermoFisher, #A-11011)

Validation

Rabbit polyclonal anti-human alpha lactalbumin has been validated for WB, ICC and IHC with reactivity to human tissues.
Goat anti-rabbit-Alexa Fluor 568 has been validated for IHC, ICC and IF. To minimize cross-reactivity, these goat anti-rabbit IgG whole antibodies have been cross-adsorbed against human IgG, human serum, mouse IgG, mouse serum, and bovine serum.
The detailed information can be obtained from the manufacturers' websites.

Eukaryotic cell lines

Policy information about cell lines

Cell line source(s)

A549 lung carcinoma cells (ATCC #CCL-185, RRID:CVCL_0023) and A498 kidney epithelial cells (ATCC #HTB-44, RRID:CVCL_1056) were obtained from ATCC.
Mouse bladder carcinoma cells (MB49, RRID:CVCL_7076) cells were provided by Sara Mangsbo, Uppsala University, Sweden.

Authentication

None of the cell lines were authenticated.

Mycoplasma contamination

Cells did not show signs of mycoplasma contamination by PCR.

Commonly misidentified lines
(See ICLAC register)

None of the cell lines used have been listed in the ICLAC register.

Human research participants

Policy information about studies involving human research participants

Population characteristics

Demographic data, morbidity and health parameters as well as tumor characteristics were recorded by the study physicians in the CRF and closely monitored by an external monitor. Population characteristics were evaluated by the study statistician. No significant differences between the treatment and placebo groups were registered in terms of age, gender, co-morbidity or tumor parameters.

Recruitment

Participants with bladder cancer were recruited at the Motol University Hospital in Prague at the Department of Urology. Inclusion and exclusion criteria were carefully defined in the study protocol. Inclusion was completed after signed informed consent.

Ethics oversight

The study was approved by the State Institute for Drug Control (SUKL) in the Czech Republic (number 273799/17-I) and the Ethics Committee of the Motol University Hospital (number EK-786/17).

Note that full information on the approval of the study protocol must also be provided in the manuscript.

Clinical data

Policy information about clinical studies
All manuscripts should comply with the ICMJEguidelines for publication of clinical research and a completedCONSORT checklist must be included with all submissions.

Clinical trial registration

EudraCT Number: 2016-004269-14 and ClinicalTrials.gov NCT03560479

Study protocol

The investigational product alpha1H is a synthetic peptide, corresponding to the alpha1 domain of α-lactalbumin, in complex with oleic acid. Alpha1H is a further development product of HAMLET, a complex between human alpha-lactalbumin and oleic acid, which has shown broad anti-tumor activity with a high degree of selectivity.
This study is a combined phase 1/2, placebo controlled, double blind study in subjects with non-muscle invasive bladder cancer awaiting transurethral resection of bladder (TURB). The study is randomized 1/1 and the subjects will receive intravesical instillation of either alpha1H (7.4 mg/mL) or placebo on 6 occasions during a period of 22 days (on days 1, 3, 5, 8, 15 and 22).
Cell shedding is quantified and characterized at each treatment occasion. The bladder tumors are characterized prior treatment and

	prior to the scheduled surgery. Remaining tumors will be removed by TURB (according to European Association of Urology (EAU) Guidelines recommendations) and tissues will be obtained for analyses.<br>A follow-up Visit will take place 30 days after the last administration of study treatment. The total study duration is 8 - 12 weeks.
Data collection	Recruitment at Motol University Hospital Prague, Czechia, from May 21st, 2018 to June 3rd 2019.<br>The randomization plan was generated centrally by an unblinded biostatistician at the Monitoring organization NEOX and was available only to unblinded study members. The randomization list linked each vial and enrolled subject with either alpha1-oleate or placebo. A randomization expert performed, documented and produced a randomization list sent to an unblinded randomization associate responsible for preparing the randomization code envelopes and a manufacturer person responsible for labelling study material.<br>The study treatment allocation list linked each study subject randomization number with a unique 6 vial code and was available to all study members. At enrollment, a randomization number was assigned to the subject on a first come first serve basis. At Visit 1 the investigator allocated the randomization number together with the unique 6 vial codes according to study treatment allocation list. Data was collected until study data lock on July 14th 2019.<br>Unblinding was done after complete data collection and Data lock on July 16th, 2019.
Outcomes	Primary Outcome Measures :<br>- Safety as Adverse Events Profile [Time Frame: From signing of informed consent (Day 1) and until End of Study (Day 52)]: Incidence of adverse events and classification in terms of severity, causality and outcome<br>- Efficacy as Cell Shedding [Time Frame: Days 1 to 22]: Change in cell shedding into urine (number of epithelial cells per mL of urine).<br>- Change from baseline in characteristics of papillary tumors [Time Frame: Prior to treatment (Baseline) and on Day 30, in connection with scheduled surgery]: The bladder tumors will be characterised by in vivo imaging during examination by cystoscopy.
